# Voltage imaging reveals hippocampal inhibitory dynamics shaping pyramidal memory-encoding sequences

**DOI:** 10.1038/s41593-025-02016-y

**Published:** 2025-07-22

**Authors:** Jiannis Taxidis, Blake Madruga, Karen Safaryan, Conor C. Dorian, Maxwell D. Melin, Zoë Day, Michael Z. Lin, Peyman Golshani

**Affiliations:** 1 Department of Neurology, David Geffen School of Medicine, University of California Los Angeles, Los Angeles, CA USA; 2 Program in Neurosciences and Mental Health, Hospital for Sick Children, Toronto, Ontario Canada; 3 Department of Physiology, University of Toronto, Toronto, Ontario Canada; 4 Picower Institute for Learning and Memory, Massachusetts Institute of Technology, Cambridge, MA, USA; 5 Department of Neurobiology, David Geffen School of Medicine, University of California Los Angeles, Los Angeles, CA USA; 6 UCLA-Caltech Medical Scientist Training Program, Los Angeles, CA USA; 7 UCSF Medical Scientist Training Program, San Francisco, CA USA; 8 Department of Neurobiology, Stanford University Medical Center, Stanford, CA USA; 9 Department of Bioengineering, Stanford University, Stanford, CA USA; 10 Greater Los Angeles Veteran Affairs Medical Center, Los Angeles, CA USA; 11 Intellectual and Developmental Disabilities Research Center, University of California Los Angeles, Los Angeles, CA USA; 12 Semel Institute for Neuroscience and Human Behavior, University of California Los Angeles, Los Angeles, CA USA; 13 Integrative Center for Learning and Memory, University of California Los Angeles, Los Angeles, CA USA

**Keywords:** Hippocampus, Neural circuits

## Abstract

Hippocampal spiking sequences encode and link behaviorally relevant information across time. How inhibition sculpts these sequences remains unclear. We performed longitudinal voltage imaging of CA1 parvalbumin- and somatostatin-expressing interneurons in mice performing an odor-cued working memory task. Unlike pyramidal odor-specific sequences that encode odor and time throughout a delay period, interneurons encoded odor delivery, but not odor identity or delay time. Odor-triggered inhibition was exerted by stable numbers of interneurons across days, with constant cell turnover, independent of task training. At odor onset, brief spiking of parvalbumin interneurons was followed by widespread hyperpolarization and synchronized theta-paced rebound spiking across interneurons. Electrophysiology, optogenetics and calcium imaging corroborated that parvalbumin interneurons silenced most pyramidal cells during odor delivery, whereas somatostatin interneurons suppressed other interneurons. The few odor-selective pyramidal cells spiked together with interneuronal post-hyperpolarization rebound. Collectively, inhibition increases the signal-to-noise ratio of pyramidal cue representations, enabling efficient encoding of memory-relevant information.

## Abstract (web summary)

Using voltage imaging, the authors show that interneurons in the hippocampus sharpen memory-encoding-activity by increasing the signal-to-noise ratio of pyramidal neuron sensory representations during working memory in mice.

## Main

The hippocampus is critical for transforming a sequence of experiences into memories, while also keeping track of their temporal relationships^1^. Sequentially activated ensembles of pyramidal cells are thought to underlie this function^1,2^. Some hippocampal pyramidal cells respond to memory-relevant sensory cues (‘cue cells’), generating sensory representations^3–6^. Other pyramidal cells (‘time cells’), fire in succession after the cue, encoding its memory as well as the time elapsed since its presentation^5,7–10^. Spiking sequences of cue cells followed by cue-specific time cells, provide a neural mechanism for linking contiguous experiences across gaps in time into a memory trace^2,11,12^.

Population activity patterns, including such spiking sequences, require the close coordination between excitation and inhibition in neuronal networks^13^. Yet, the role of inhibitory neurons in sculpting memory-encoding hippocampal dynamics is unclear. Inhibition in the hippocampus is regulated by a diverse array of GABAergic interneurons, divided into subtypes with distinct anatomical organization, connectivity, firing patterns and genetic profiles^14,15^. Two major subtypes are parvalbumin-expressing (PV) and somatostatin-expressing (SST) interneurons, which mostly target perisomatic areas and dendrites of pyramidal cells, respectively. Perisomatic PV inhibition controls the timing of pyramidal output^16^ and coordinates rhythmic population activity^17–19^. Dendritic SST inhibition gates the integration of proximal and distal dendritic inputs on pyramidal cells^20^ and ultimately their gain (their output relative to their input)^21^. The interplay between pyramidal cells, PV and SST interneurons shapes hippocampal spiking in vivo and controls behavior (reviewed elsewhere^22–25^); however, this interplay has rarely been studied outside of spatial navigation. As a result, the role of PV and SST neurons in sculpting memory-encoding spiking sequences, combining cue cells and time cells, remains unknown.

Exploring the role of inhibition in memory processes requires monitoring activity from identified interneuron subtypes with high temporal resolution, to reliably record changes in their high firing rates. It also requires tracking the same cells across memory-relevant timescales of multiple days. Electrophysiology does not allow combined cell-type specificity and multiday cell tracking, whereas calcium imaging cannot capture the fast-spiking dynamics of interneurons. Moreover, neither calcium imaging nor extracellular electrophysiology captures subthreshold membrane potentials which contain valuable information on synaptic inputs. Fast frame-rate voltage imaging of genetically encoded voltage indicators (GEVIs)^26,27^ overcomes these barriers and captures action potentials as well as subthreshold membrane potentials. It also allows imaging of the same cells for multiple days^28^.

We employed kHz-rate voltage imaging to investigate the spiking and membrane potential dynamics of PV and SST hippocampal interneurons during an odor-cued working memory task. We have previously described pyramidal sequences in this task, composed of ‘odor cells’, encoding specific odor cues, followed by odor-specific time cells throughout an ensuing delay period, encoding the preceding cue as well as delay time^5^. Here we found that CA1 PV and SST interneurons are synchronously activated by the odor delivery, but not in an odor-selective manner and irrespective of task engagement by the mouse. By supplementing our voltage imaging with optogenetic manipulations during electrophysiological recordings, as well as two-photon calcium imaging, we demonstrate that odor-timed inhibition increases the signal-to-noise ratio of pyramidal odor cells, allowing for efficient encoding of cue information during memory activation.

## Results

### In vivo voltage imaging of PV and SST cells during DNMS

**Fig. 1:**
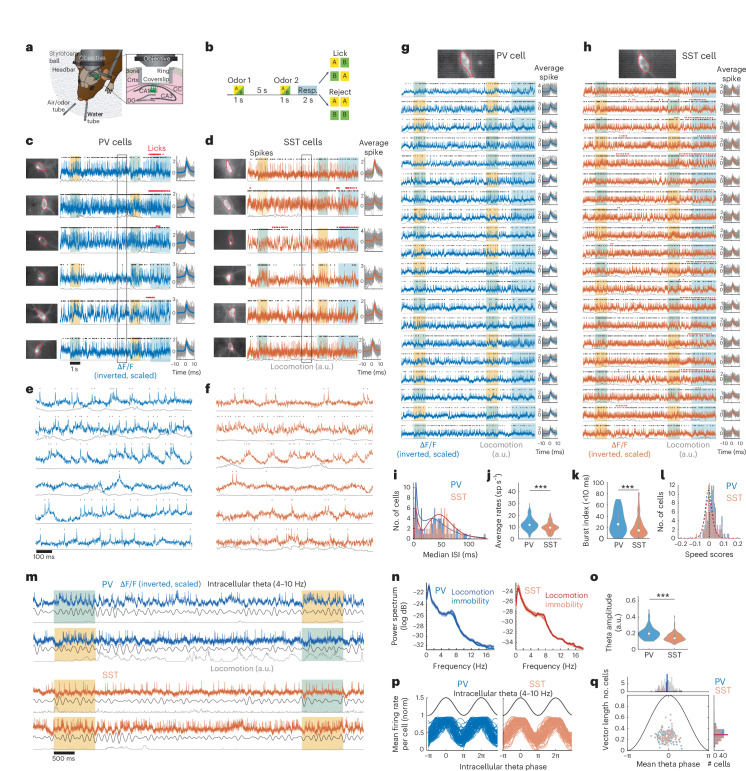
In vivo voltage imaging of CA1 PV and SST interneurons during DNMS. **a**, Behavioral and experimental setup. Crtx, cortex; CC, corpus callosum. **b**, Schematic of the DNMS trial. Yellow indicates ‘odor A’. Green indicates ‘odor B’. Blue shows response window for assessing licking. **c**,**d**, Example traces from PV (**c**) and SST (**d**) interneurons during a DNMS trial. Average FOV with outlined region of interest (ROI) (left). Inverted ΔF/F, scaled by maximum value (middle). Black dots show detected spikes; red indicates licks; color boxes show odor cues and response window as in **b**. Gray traces indicate locomotion. Peri-spike ΔF/F during all (gray) and average (thick line) action potentials in the trial (right). **e**,**f**, Expanded traces from boxes in **c** and **d**. **g**,**h**, Example PV (**g**) and SST (**h**) interneuron recorded during 20 continuous DNMS trials, plotted as in **c** and **d**. **i**, Histogram of median interspike interval per cell and estimated probability density (solid lines). **j**,**k**, Mean firing rates (**j**) and burst index (**k**) in PV versus SST cells; *P* = 4.28 × 10^−5^, 8 × 10^−4^; two-sided Wilcoxon rank-sum test (WT). **l**. Histogram of mean speed score per cell. Dashed lines show mean shuffle baseline per cell group. **m**, Example raw and 4–10 Hz theta-bandpassed ΔF/F from PV and SS cells. **n**, Mean ± s.e. power spectral density of each cell group during motion versus immobility. No significant differences exist (*P* > 0.05; WT per frequency; false discovery rate (FDR)). **o**, Mean intracellular theta amplitude for PV versus SST cells; *P* = 1.47 × 10^−9^; two-sided WT. **p**, Mean firing rate of each PV and SST cell, normalized to maximum, over the intracellular theta cycle (black). **q**, Mean strength (vector length) versus preferred phase of theta modulation per cell. Black shows intracellular theta cycle. Distributions of preferred phase (top) and modulation strength (right) are similar for both cell groups (*P* > 0.05; parametric Watson–Williams multisample test and WT, respectively). Lines indicate distribution means. In all violin plots throughout figures, dots are median values, boxes are 25% and 75% quartiles and whiskers show 1.5× interquartile range. **i**–**l** and **n**–**q** contain all cells pooled (*n* = 107 PV, 93 SST cells).

To capture fast-scale spiking and membrane potential dynamics of interneurons, we expressed the GEVI ASAP3 in hippocampal PV or SST interneurons. Adult PV-Cre and SST-Cre mice (*n* = 5 mice in each group) were virally transfected in right dorsal CA1 with Cre-dependent ‘ASAP3’ (AAV8-ef1α -DiO-ASAP3-Kv)^28^ and implanted with an imaging window above CA1 and a metal headbar for head fixation (Fig. 1a). Mice were later water-deprived and trained on an olfactory delayed non-match-to-sample (DNMS) working memory task, while head-fixed on a treadmill (Fig. 1a)^5,29^. Each trial consisted of two odor cues of 1 s each, with a 5-s delay between them. Mice were trained to lick a lickport to release water rewards only if the two odors did not match, and refrain from licking if they matched (Fig. 1b). Odor cues were either isoamyl acetate (‘odor A’) or pinene (‘odor B’) and were delivered in random combinations. Licking in odor-match trials was not punished, but no reward was delivered. Performance was quantified as the percentage of correct hits and rejections. We have previously shown that, after a shaping period, mice learn to perform DNMS within ~5–6 days, using working memory^5^.

We first conducted voltage imaging of PV or SST interneurons for two imaging sessions (separate days) while mice were naive to the task and passively smelled the odor cues. We then trained mice on the DNMS task (~10–14 days total) and resumed voltage imaging after they were well trained (Methods). In trained imaging sessions, PV-Cre mice reached a higher performance than SST-Cre mice (71.9 ± 1.9% versus 62.1 ± 1.8% per recording (mean ± s.d.), respectively. *P* = 3.51 × 10^−4^, two-sided *t*-test, *n* = 104 PV, 84 SST recordings), though the same training protocol was used throughout. Note that, unless otherwise stated, ensuing findings are independent of performance levels. Imaging took place with a custom-made epifluorescence microscope. The power of the excitation LED was set lower for SST cells, which lie mostly in stratum oriens and were thus closer to the coverslip (119.9 ± 42.7 and 100.1 ± 27.4 mW mm^−2^) for PV and SST recordings, respectively (*n* = 104 PV, 140 SST recordings. *P* = 1.6 × 10^−5^; two-sided *t*-test). The field of view (FOV) was set to 88 × 44 µm (64 × 32 pixels), which allowed for image acquisition at 1,000 frames per second, and in most cases encompassed a single cell. Recordings captured action potentials and subthreshold membrane potential fluctuations (Fig. 1c–f). We were able to record activity across multiple trials (up to 48 from a single cell), before clear spike waveforms and subthreshold dynamics were attenuated due to GEVI photobleaching (Fig. 1g,h). Overall, we recorded 107 PV cells and 93 SST cells (21.4 ± 9.1 and 18.6 ± 12.6 per mouse) in 104 and 90 recordings, respectively, over 16.2 ± 6.5 trials per PV cell and 14.7 ± 8.5 trials per SST cell. In naive sessions, 15.4 ± 6.8 trials and 14.4 ± 7.4 trials were recorded per PV and SST cell, respectively, in 31 and 35 videos. In trained sessions, 16.5 ± 6.5 and 14.9 ± 9.2 trials were recorded in 73 and 55 videos, respectively. Recordings were processed using the Volpy automated pipeline in Python^30^.

**Extended Data Fig. 1:**
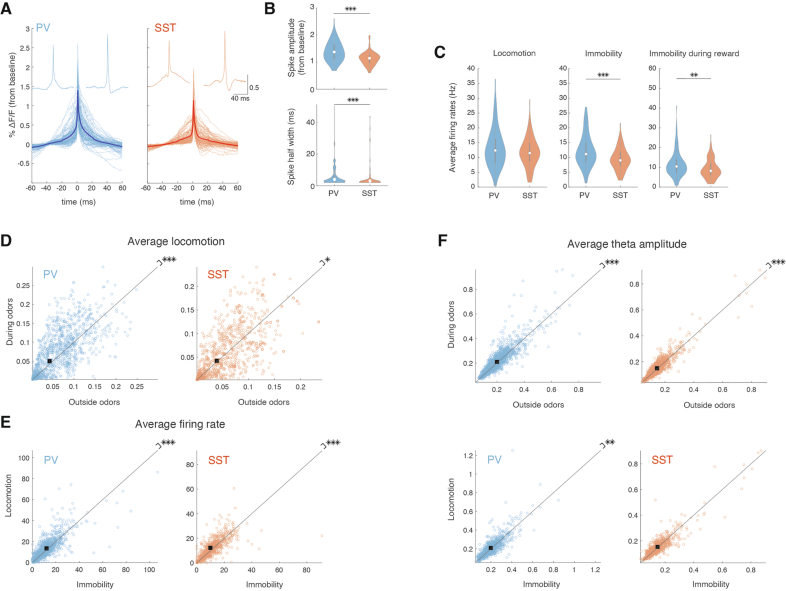
Spiking, intracellular theta and locomotion in PV and SST cells. **a**. Average action potential waveform and individual examples in PV and SST cells. **b**. Mean spike amplitude (top) and spike width (bottom) was higher in PV versus SST cells (P = 1.37 × 10^−7^ and 5.45 × 10^−6^ respectively; two-sided Wilcoxon test – ‘WT’). 1 PV outlier removed from **a** and **b** (bottom) for plotting clarity. **c**. Mean firing rates across locomotion, immobility, and immobility during the reward window in PV vs SST cells (only cells with both locomotion and immobility segments used for locomotion and immobility comparisons: n = 98 vs 95 cells. For third comparison all cells included: 107 vs 93 cells. From left: P = 0.122, 0.0001, 0.0048; two-sided t-test). **d**. Mean locomotion (a.u.) during odor delivery versus outside odor delivery in each trial in PV-Cre and SST-Cre mice. Black rectangle: Average across trials was significantly higher during odors in both cell groups (n = 1730 trials, P = 6.36 × 10^−21^ for PV; n = 1390, P = 0.014 for SST; paired-sample two-sided t-test). **e**. Mean firing rates of PV and SST cells during locomotion versus immobility segments in each trial. Black rectangle: Average across cells and trials was significantly higher for locomotion in both cell groups (only trials with both motion and immobility segments included, excluding time bins around trial onset and offset – see Methods. n = 985, P = 5.19 × 10^−10^ for PV, n = 746; P = 0.0003 for SST; paired-sample two-sided t-test). **f**. Top: Mean intracellular theta amplitude (a.u.) during odor delivery versus outside odor delivery per trial (n = 1730 trials, P = 4.89 × 10^−17^ for PV; n = 1390, P = 4.21×10^−11^ for SST). Bottom: Mean intracellular theta amplitude during locomotion versus immobility segments in each trial (only trials with both motion and immobility segments included, excluding time points around trial onset and offset – see Methods. n = 1005, P = 0.0023 for PV; n = 763, P = 0.715 for SST; paired-sample two-sided t-test).

At higher signal-to-noise ratios, optically reported action potentials of PV interneurons were higher in amplitude and wider in duration than those of SST interneurons, and an after-hyperpolarization was often recorded in both cell groups (Extended Data Fig. 1). PV interneurons exhibited lower interspike intervals than SST interneurons (Fig. 1i), yielding higher average firing rates and burst index (Fig. 1j,k)^16^. The higher firing rates in PV interneurons were observed in immobility segments, including immobility during reward delivery, but not during locomotion on the treadmill (Extended Data Fig. 1).

Locomotion was higher during odors in PV-Cre and SST-Cre mice (Extended Data Fig. 1). Average firing rates were higher during locomotion in both PV and SST interneurons (Extended Data Fig. 1), even when excluding odor segments; however, correlations between firing rates and locomotion (‘speed scores’; Methods) remained low, with 79.4% PV and 85% SST cells having below-chance speed scores (Fig. 1l).

In both PV and SST interneurons, ΔF/F traces and spiking often exhibited pronounced theta rhythmicity (Fig. 1m). This was reflected in 4–10 Hz peaks in the average power spectra of ΔF/F, after action potential removal (de-spiking; Methods), during motion and immobility segments (Fig. 1n). Intracellular theta amplitudes were higher for PV than SST interneurons on average (Fig. 1o). Spiking was strongly phase-locked to intracellular theta peaks, similarly for PV and SST interneurons (Fig. 1p–q). During odor presentation, intracellular theta increased in both PV and SST interneurons (Extended Data Fig. 1). During locomotion, intracellular theta increased in PV but not SST interneurons (Extended Data Fig. 1), suggesting that SST theta may be more strongly driven by odors than locomotion.

Collectively, in vivo fast-rate voltage imaging, with the ASAP3 GEVI, reliably captured spiking and membrane potential dynamics of PV and SST interneurons during the DNMS task and revealed an association between the intracellular theta oscillations in these interneurons with odor presentation.

### PV–SST cells encode odor delivery not odor identity or delay time

During the DNMS task, CA1 pyramidal cells form spiking sequences comprising ‘odor cells’ that spike during a specific odor, followed by ‘time cells’, each spiking at a particular time point in the delay after a specific odor^5^. We asked whether interneurons form similar odor-specific spiking sequences.

**Fig. 2:**
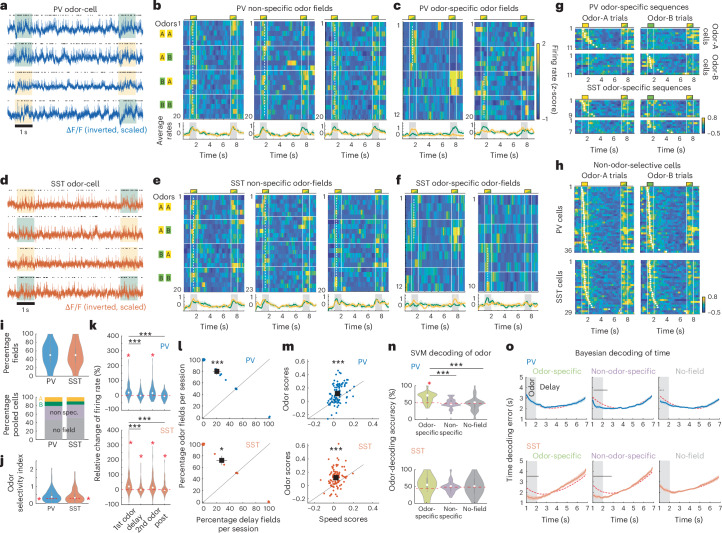
Interneurons encode cue presentation, not odor or delay time. **a**, Example traces from PV odor field, displayed as in Fig. 1. **b**, Example PV odor fields, encoding both odor A and odor B presentation (left is same cell as in **a**). Each row is the neuron’s z-scored firing rate during a trial, with trials stacked by odor combination (left). Vertical lines show odor delivery (trial layout shown on top). Dashed line indicates firing field time bin. Mean ± s.e. rate over all odor A (yellow) and odor B trials (green) (bottom). **c**, Same for odor-specific PV interneurons encoding odor A (left) or odor B (right). Dashed lines cover preferred-odor trials. **d**–**f**, Same as **a**–**c** for SST interneurons. **g**, Average *z*-scored firing rates of odor A-specific (top row) and odor B-specific (bottom row) PV cells over odor A (left) and odor B (right) trials, stacked by field time bin (dots) (top). Same for SST cells (bottom). **h**, Average firing rates of non-odor-specific PV (top) and SST cells (bottom) over odor A and odor B trials. **i**, Percentage field cells per mouse per session (*P* = 0.78, two-sided WT; *n* = 28 PV, 28 SST sessions) (top). Mean cumulative % of odor-specific, non-odor-specific and no-field cells (bottom). **j**, Odor selectivity (absolute values). *P* = 0.39, two-sided WT (*n* = 106, 93 cells, one outlier removed). Dashed lines show chance selectivity. Red *P = 7.32 × 10^−6^, 0.0026; two-sided WT against chance. **k**, Mean firing rate change from baseline, during the first odor, the delay, the second odor and the response window in field cells (*n* = 107 PV, 91 SST cells). ***P_1st odor-delay_ = 6 × 10^−7^, 7.3 × 10^−5^ (PV, SST); *P*_1st odor-post_ = 4.2 × 10^−9^, 9.8 × 10^−5^; *P*_1st odor-2nd odor_ = 0.065, 0.323; two-sided WT. Red *P < 0.05, right-tailed *t*-test against zero; FDR corrected. **l**, Percentage odor versus delay fields per session. Small jitter added for clarity. Square indicates distribution means. ***P = 5.58 × 10^−5^, *P = 0.012; paired-sample two-sided *t*-test. **m**, Odor scores versus speed scores (*n* = 107 PV, 93 SST cells). ***P = 4.48 × 10^−9^, 1.58 × 10^−9^, paired-sample two-sided *t*-test. **n**, Odor-decoding accuracy with SVM decoders trained on odor-specific cells (*n* = 21 PV, 12 SST), non-odor-specific cells (*n* = 34, 25) or no-field cells (*n* = 39, 27), during odor presentation. ***P < 0.001, two-sided WT, FDR corrected. Dashed lines show mean shuffle baselines. Red *P = 0.0003, right-tailed WT against chance baseline; FDR corrected. **o**, Mean ± s.e. time-decoding error (absolute) across the odor-delay interval, with Bayesian decoders trained as in **n**. Dashed lines indicate mean shuffle baselines. Black bars show *P* < 0.05; two-sided WT, FDR corrected.

As pyramidal sequences can also be observed in untrained mice, we pooled data from all sessions, including naive exposure to DNMS trials. Analysis hereafter focuses on the period covering the first odor presentation and the ensuing delay in each trial (‘odor-delay interval’). Interneurons fired mostly continuously, but many cells increased their firing during odor presentation (Fig. 2a,d). Many interneurons exhibited significant temporal tuning (‘firing fields’; Methods) during the odor presentation but independently of the odor identity (Fig. 2b,e). Unlike pyramidal cells^5^, few interneurons had odor-specific fields (Fig. 2c,f), yielding sparse odor-specific sequences when pooled across mice and sessions (11 PV and 8 SST cells were odor A-specific; 10.3% and 8.6% of all cells in each group. Eleven PV and 7 SST cells were odor B-specific (10.3% and 7.5%, respectively; Fig. 2g). In contrast, non-odor-specific fields were found in 36 PV and 30 SST cells (33.6% and 33.2% of all cells in each group; Fig. 2h). PV and SST cells had similar percentage of detected fields per session and ratios of odor A-specific, odor B-specific and non-odor-specific fields (Fig. 2i). Average odor selectivity was also similar between PV and SST cells and was slightly but significantly above chance for both groups (Fig. 2j). In total, 54.2% of all PV cells and 48.4% of SST cells had a significant field within the odor-delay interval (‘field cells’), whereas 45.8% and 51.6% cells showed no significant fields (‘no-field cells’). Firing rates of PV and SST field cells were higher during odors than delay period or response window and increased similarly for the first and second odor in a trial (Fig. 2k). During delay, PV rates returned to baseline, whereas SST ones remained higher (Fig. 2k).

**Extended Data Fig. 2:**
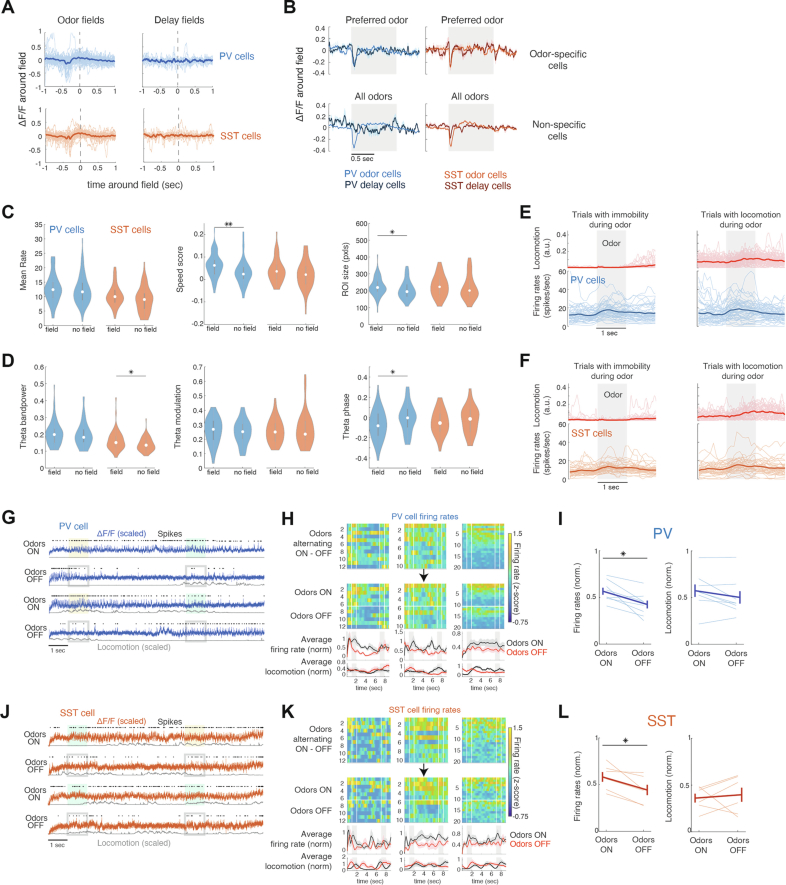
Spiking and subthreshold properties of field cells and their correlation with locomotion and odors-OFF. **a**. Average de-spiked ΔF/F traces of odor-field cells (left) and delay-field cells (right) around their field time point for individual cells (thin lines) and averaged across cells (thick). **b**. Average de-spiked ΔF/F traces of PV and SST odor-field and delay-field cells during the first odor delivery (gray box). Top: Odor-specific cells. Bottom: Non-specific cells. No significant differences observed at any time point in the odor (P > 0.05, WT). **c**. Mean firing rates across trials, speed scores and ROI sizes (number of pixels of each ROI) for PV and SST field cells (n = 58 PV, 45 SST cells) versus non-field cells (n = 49 PV, 48 SST). * P = 0.0399, ** P = 0.001, all other P > 0.05, two-sided WT. **d**. Mean theta amplitude, strength of theta modulation of spiking (vector length) and theta phase (measured in 1/π, 0 corresponds to theta peaks for field cells versus non-field cells, plotted as in **c**. P = 0.0329 for SST theta power, P = 0.014 for PV theta phase, all other P > 0.05; parametric Watson–Williams test for theta phase and two-sided WT for power and modulation. **e-f**. Mean firing rates of individual PV (**e**) and SST (**f**) odor-field cells (thin lines) and averaged across cells (thick) during odor delivery for trials with immobility (left) and locomotion (right) during odors. Corresponding locomotion traces shown on top row. **g**. Example traces from a PV cell across 4 trials, displayed as in Fig. 1c. Gray boxes: Odor cue windows in trials where the odor was not delivered. **h**. Top row: Firing rates from 3 PV cells across trials with alternating odors ON and OFF (scaled by maximum average rate across odor/delay bins). Second row: Same firing rates rearranged over all odor ON and OFF trials (separated by white line). Third row: Mean ± SE firing rates across odor ON and OFF trials (black and red respectively). Bottom row: Average locomotion in the same trials. **i**. Mean ± SE firing rates (averaged across all odor/delay bins and all corresponding trials) of each cell (left) and corresponding mean locomotion (right) in odor ON versus odor OFF trials. n = 8 cells; P = 0.0108, 0.083; paired-sample two-sided t-test. **j–l**. Same as G-I for SST cells. In panel **l**: n = 6 cells; P = 0.0184, 0.683; paired-sample two-sided t-test.

Most fields were observed during odor delivery (‘odor fields’; Fig. 2h). Firing rates were rarely tuned to delay time points, resulting in very sparse ‘delay fields’ (Fig. 2l; 9.3% PV and 12.9% SST cells had delay fields. 50% and 58.3% of those were within 1 s from odor offset). Odor fields, but not delay fields, coincided with a depolarized ΔF/F, whereas during odors both field types yielded similar subthreshold traces (Extended Data Fig. 2).

PV field cells had similar average firing rates but a larger soma and stronger correlation with locomotion than no-field PV cells, suggesting that they may represent distinct PV subtypes. PV field cells also spiked at an earlier intracellular theta phase but had similar theta power and theta spiking modulation with no-field ones. In contrast, SST field cells showed no differences with no-field cells in these metrics, except for stronger intracellular theta (Extended Data Fig. 2).

We tested whether odor fields were triggered by locomotion, which was higher during odors. Odor-field cells had similar firing rates in trials when the mouse was immobile versus locomoting during odor delivery (Extended Data Fig. 2). Furthermore, odor-field spiking was more correlated with odor delivery than locomotion (Fig. 2m), with 55.1% and 58.1% of PV and SST cells having significant odor scores (similarly to speed scores; Methods), compared to 20.6% and 15% for speed scores, respectively. Therefore, spiking was more strongly correlated with odor delivery than locomotion.

Furthermore, odor fields were not triggered by clicking of the odor air valve. In some recordings, the odor airflow was turned off on alternate trials while all valves remained operational. PV and SST spiking was reduced during odor OFF trials, often throughout the delay interval. Average PV and SST firing rates were significantly lower during OFF trials, whereas average locomotion was unaffected (Extended Data Fig. 2), supporting that odor fields are generated by odor delivery rather than clicking or locomotion.

To confirm most interneurons were not odor selective, we trained binary support vector machine (SVM) classifiers on each cell’s activity over the first odor. As expected, SVM classifiers trained on odor-specific PV cells, decoded odor identity better than chance, and better than those trained on non-odor-specific cells or no-field cells, both of which performed at chance levels, as did all SST-trained classifiers, including those trained on odor-specific SST cells (Fig. 2n). Classifiers trained on the entire odor-delay interval, instead of the first odor, performed at chance levels (*P* > 0.05 one-sided WT; compared to shuffle baseline).

We also trained Bayesian decoders on each cell’s firing rate to decode time throughout the odor-delay interval. The prevalence of odor fields resulted in efficient time-decoding during odors but chance levels during the delay period for most cell groups (Fig. 2o). Bayesian-based odor-decoding was at chance levels in all cases (*P* > 0.05 compared to shuffle baseline for all bins).

Collectively, these findings demonstrate that, unlike odor-selective spiking sequences of pyramidal cells throughout the odor-delay interval, PV and SST interneurons encode mostly odor delivery, not delay time, and are not odor-selective.

### Steady-state inhibition across days independent of training

Pyramidal sequences combine stable odor fields with highly dynamic delay fields across days which increase in number as mice learn the DNMS task^5^. We next examined whether interneuronal fields show similar stability and learning-related dynamics.

**Fig. 3:**
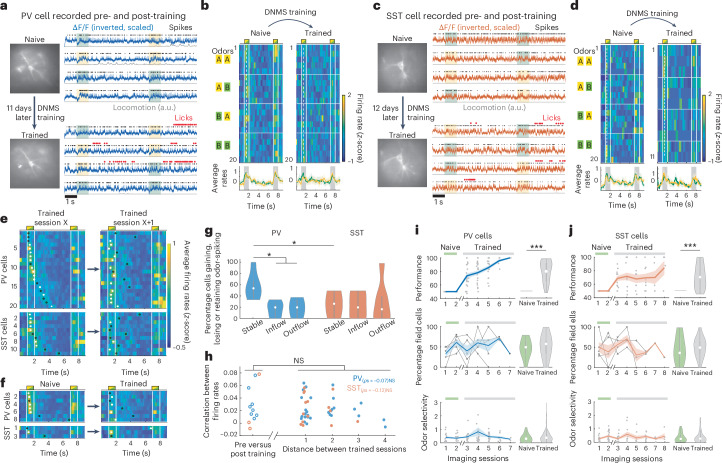
Interneuron stability across days and DNMS training. **a**, Example PV cell during four DNMS trials before and after DNMS training. Expanded FOV (left). **b**, Firing rates per trial and odor-specific averages across the two recording sessions, plotted as in Fig. 2. **c**,**d**, Same as **a**,**b** for an SST cell. **e**, Average firing rates across trials for pooled PV (top) and SST cells (bottom) on any session X and following session X + 1. Cells stacked by maximum rate time bin on session X. Cells recorded for >2 sessions shown independently for each consecutive pair. White dots show significant fields. Black circles show nonsignificant firing peaks. **f**, Same for last naive session versus first trained session. **g**, Percentage cells recorded across two consecutive sessions that retained odor spiking (‘stable’), moved their firing peak into (‘inflow’) or out of the odor delivery time bins (‘outflow’). *n* = 7 and eight session pairs for PV and SST; *P_stable-inflow_ = 0.013; *P*_stable-outflow_ = 0.016; *P*_stable-stable_ = 0.049; all other *P* > 0.05; two-sided WT; FDR corrected. **h**, Mean cross-correlation of a cell’s firing rates across all trials between two sessions, as a function of distance between the sessions. Pre-Post, same between last naive versus first trained session (*P* > 0.05, two-sided WT for both cell groups). Small jitter added for clarity. *ρ*_S_, Spearman correlation between distance of trained sessions and firing rate correlations (*P* > 0.05 for both cell groups). **i**,**j**, Performances of PV-Cre (**i**) and SST-Cre mice (**j**) per recording (dots) and mean ± s.e. per day (lines) (top). Naive and trained sessions indicated on top. Note that multiple training days exist between the two groups. Mean ± s.e. (middle). Percentage field cells over all PV (**i**) and SST cells (**j**) per session. Lines indicate individual mice. Mean ± s.e. odor selectivity of cells (absolute values) (bottom). Pooled distributions for naive versus trained sessions (right). For PV, from top, *n* = 31 versus 73 recordings, 10 versus 18 sessions, 31 versus 75 cells; ***P = 2.24 × 10^−4^, two-sided WT. For SST, from top, *n* = 35 versus 49 recordings, 10 versus 15 sessions, 36 versus 51 cells: ***P = 6.58 × 10^−10^, two-sided WT.

**Extended Data Fig. 3:**
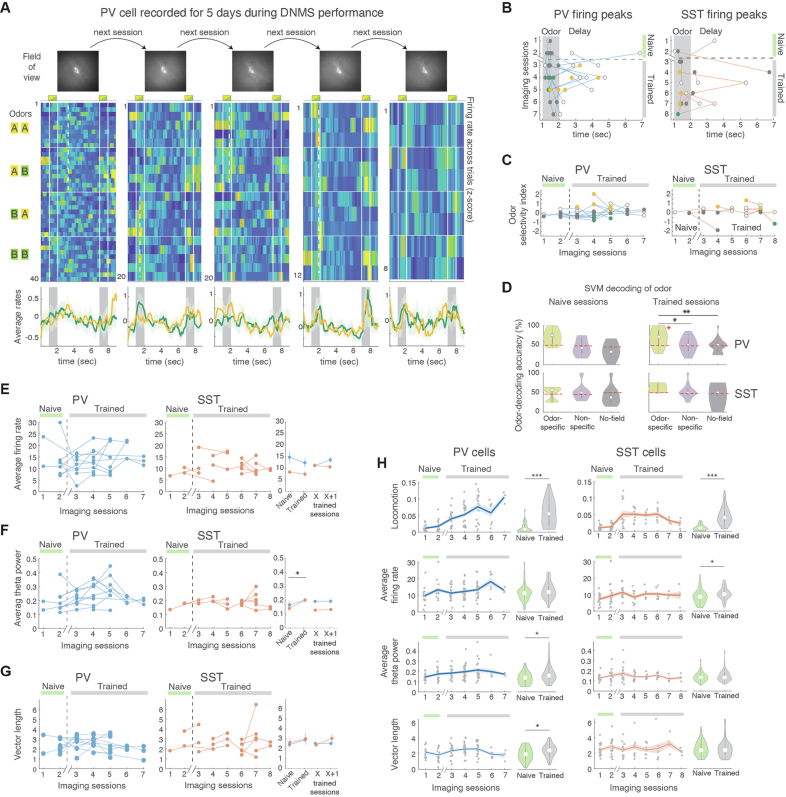
Interneuron firing properties remain overall stable across days and over training. **a**. Example PV cell recorded for 5 consecutive imaging sessions, plotted as in Fig. 3. Dashed lines: significant field over a specific odor (line covers corresponding trials) or through both odors (Day 4). **b**. Progression of firing peak time bins across days for PV (left) and SST cells (right) recorded across multiple sessions. Gray: Non-odor-specific fields. Yellow, green: odor A- or odor B-specific fields, respectively. Open circles: nonsignificant peaks. Lines connect a cell’s progression. **c**. Progression of odor selectivity index across days, displayed as in **b** (sessions on *x* axis). **d**. Odor-decoding accuracy of SVM decoders trained on odor-specific cells, non-odor-specific cells, or no-field cells, during odor-presentation, in naïve (top) and trained sessions (bottom), plotted as in Fig. 2n. **e–g**. Progression of average firing rate (**e**), theta power (**f**) and phase locking strength (mean vector length; **g**) across all trials, as in **c** (field-type not displayed). Right: Mean ± SE for last naïve vs first trained session and for every X vs X + 1 trained session. * P = 0.0068, paired-sample two-sided t-test (distributions of PV and SST cells were pooled for this comparison; n = 11. P > 0.05 for all other comparisons). **h**. From top: Evolution of mean locomotion per session, mean firing rate per cell over all trials, theta power and theta modulation of spiking (vector length) per cell across PV-Cre mice and PV cells (left) or SST-Cre mice and SST cells (right), plotted as in Fig. 3i, j. For PV: n = 31 vs 73 recordings, 31 vs 76 cells; from top: P = 9.44 × 10^−10^, 0.218, 0.0146, 0.0152; two-sided WT. For SST: n = 35 vs 49 recordings, 36 vs 51 cells; from top: P = 1.23 × 10^−11^, 0.0123, 0.466, 0.543 for SST.

We tracked a subset of interneurons across multiple imaging sessions (different days) to assess changes across days or between naive and trained states. Fifteen PV cells and 11 SST cells (from four out of five PV-Cre and SST-Cre mice, respectively) were recorded over at least two separate days. To ensure that a cell was matched to a previous recording, we briefly captured an extended FOV (352 × 352 µm at < 100 Hz frame rate) containing features around the cell body that were used as reference. We recorded a cell for up to five consecutive sessions (Extended Data Fig. 3) while mice performed the DNMS task, as well as some cells before and after DNMS training (Fig. 3a,c).

Interneuron firing fields tended to remap across days. The firing peak of a cell could become a significant field or lose significance, shift along the time axis or switch between being odor-specific or nonspecific, resulting in odor selectivity that fluctuated across days (Extended Data Fig. 3). Of note, some cells exhibited the same odor field in a naive and a trained session, weeks apart (Fig. 3b,d). Moreover, PV odor fields were often retained between a trained session X and the next session X + 1, whereas most PV delay fields had turned to odor fields (Fig. 3e). Similarly, three out of five PV odor fields, recorded in a naive session, were retained in the first trained session (Fig. 3f). SST fields exhibited higher instability overall. We quantified the turnover of odor fields across days by the percentages of cells in a session that retained a field from the previous session (‘stable’) or gained one (‘inflow’) or lost their field in the next session (‘outflow’). There were significantly more stable PV odor fields than inflowing or outflowing ones. Stable SST fields were more rare than PV ones and did not differ from inflow or outflow rates (Fig. 3g).

Beyond fields, we assessed a cell’s spiking similarity across any two sessions by computing the average correlation of its firing rates across all pairs of trials between the two sessions. Correlations were low and they did not differ across trained sessions compared to pre- versus post-training for either PV or SST interneurons (Fig. 3h). Moreover, there was no relationship between correlations and the distance between imaging sessions (Fig. 3h). These findings corroborate an overall random change in interneuron firing activity between days, which was not affected by learning the DNMS task.

We also searched for any learning-related changes in the collective activity of recorded cells. Unlike pyramidal delay fields that increase in number with learning, we found stable percentages of PV and SST fields across days, as well as pre- versus post-training, even though performance improved across trained sessions (Fig. 3i,j). Average odor selectivity also remained stable (Fig. 3i,j), corroborating our observation in the multiday-tracked cells. As a result, SVM odor-decoding accuracy from field cells across only naive sessions or only trained sessions, yielded similar results to the decoding accuracy across all sessions pooled (Extended Data Fig. 3).

Finally, locomotion on the treadmill increased post-training, but seemed to minimally affect interneuron firing, with SST firing rates, but not PV ones, showing a small increase post-training (Extended Data Fig. 3). In PV cells, intracellular theta power and phase locking of spikes increased post-training. A post-training increase in theta power was also observed in multiday-tracked cells when pooling PV and SST cells, but it remained stable across trained sessions (Extended Data Fig. 3).

Collectively, our findings suggest steady-state inhibition, exerted by a stable number of interneurons over days, with fluctuating spiking modulation and irrespective of participation to the DNMS task.

### PV–SST hyperpolarization at odor onset resets intracellular theta

Little is known about subthreshold dynamics of interneurons in vivo, even though they contain information on behaviorally relevant synaptic inputs. We thus focused on the subthreshold membrane potential from PV and SST interneurons.

**Fig. 4:**
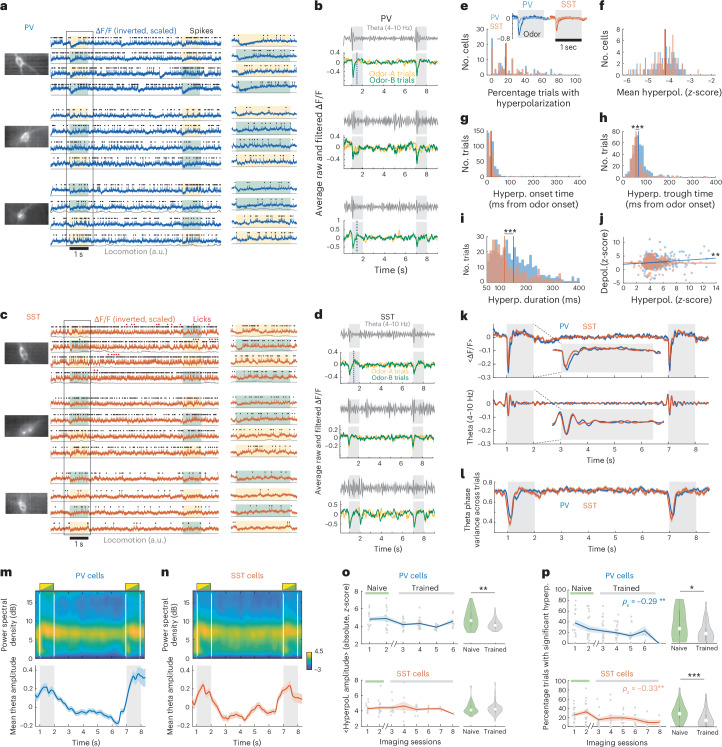
A hyperpolarization during odor onset resets intracellular theta. **a**, Example trials from three PV cells (from three different mice) with odor-triggered hyperpolarization traces. Average FOV (left). ΔF/F, detected spikes and locomotion during four DNMS trials, displayed as before (middle). Expanded traces within box (right). **b**, Trial-average theta-bandpassed ΔF/F (gray) and odor A (yellow) and odor B (green) average ΔF/F across all corresponding trials, for the three cells shown in **a**. **c**,**d**, Same as **a**,**b** for three example SST cells (from three different mice). Licks shown for one trained session. **e**, Percentage of trials with a significant hyperpolarization per cell. Lines indicate median values (*P* = 0.9; WT). Insets show average odor-triggered ΔF/F over trials with significant and nonsignificant hyperpolarization. **f**, Distribution of mean amplitude of ΔF/F hyperpolarization per cell (z-score-scaled over baseline of 0.5 s before odor onset; *P* = 0.98; WT). **g**–**i**, Distribution of hyperpolarization onset time (**g**), time of minimum ΔF/F (**h**) and hyperpolarization duration (**i**). Distributions truncated at 400 ms for clarity. *P* = 0.06, 2.4 × 10^−4^, 6.3 × 10^−4^; two-sided WT; FDR. **j**, Amplitude of maximum depolarized versus hyperpolarized ΔF/F during odor presentation. Lines show least square fit for each cell type. *P* = 0.004, 0.901 (PV, SST); F-test. One outlier removed for plotting clarity. **k**, Trial-average ΔF/F (top) and theta-bandpassed ΔF/F (bottom) for PV and SST cells. Insets show zoomed in segments around the first odor. **l**, Variance of theta phases across all trials. **m**,**n**, Average ΔF/F power spectral density for PV (**m**) and SST cells (**n**) across trials (top). Mean ± s.e. ΔF/F theta amplitude (bottom). **o**, Mean ± s.e. hyperpolarization amplitude across sessions and naive versus trained session averages (right) for PV and SST cells (*n* = 26 versus 57 PV recordings and 33 versus 36 SST). *P* = 0.0068, 1 (PV, SST); two-sided WT. **p**, Same for rate of hyperpolarization occurrence (*n* = 31 versus 76 PV recordings and 36 versus 51 SST). *P* = 0.022, 0.0005 (PV, SST), two-sided WT*. ρ*_S_, Spearman correlation with imaging sessions, *P* = 0.0027, 0.0019 (PV, SST), random permutation test.

During odor onset, a prominent negative deflection in the interneurons’ de-spiked ΔF/F across both odor A and odor B trials, signified a hyperpolarization of PV and SST interneurons (Fig. 4a,c), observed across multiple cells and trials and followed by a depolarization during odor spiking (Fig. 4b,d). Similar negative deflections could be seen during the delay but these did not occur in a systematic manner (Fig. 4a,c). A significant hyperpolarization (Methods) occurred in a similar percentage of trials in PV and SST interneurons (22.5 ± 21.5% versus 21.7 ± 18.9%) and was completely absent in 22.4% PV and 19.3% SST cells (Fig. 4e). During hyperpolarization, average de-spiked ΔF/F deflections were similar between PV and SST cells (−4.8 ± 1.7 versus −4.4 ± 1.2 s.d. from the pre-odor baseline; Fig. 4f). Hyperpolarization onset times were also similar (20 ± 20 ms versus 23 ± 60 ms after odor onset; Fig. 4g) but the hyperpolarization peaked slightly later in PV than SST cells (81 ± 70 ms versus 83 ± 97 ms after odor onset (due to more outliers in SST cells) but with medians at 68 ms versus 57 ms; Fig. 4h) and lasted longer (194 ± 166 ms versus 187 ± 203 ms after odor onset; Fig. 4i). The hyperpolarization amplitude correlated with the ensuing depolarization during the odor in PV cells only (Fig. 4j).

**Extended Data Fig. 4:**
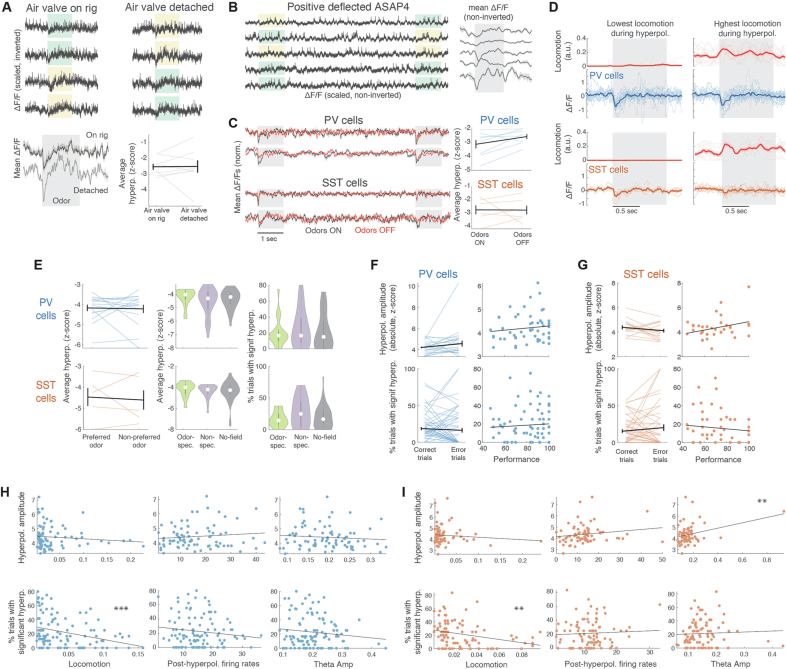
Odor-onset hyperpolarization is not artifactual and is reduced in PV cells after training. **a**. Example traces across odor cues from a cell and average ΔF/F ± SE (bottom) when the odor air valve is on the rig versus detached. Bottom right: Mean hyperpolarization (ΔF/F minimum, z-score-scaled over baseline of 0.5 sec before odor onset) in the two conditions (n = 8, P = 0.95, paired-sample two-sided t-test). **b**. Top: Example traces from an SST cell expressing the positively deflected ASAP4 GEVI and average traces from 5 SST cells over the first odor cue (right). ΔF/F traces were not inverted, yet odor-onset deflections still indicate a hyperpolarization. **c**. Average traces from two example PV (top) and two example SST cells (bottom) across trials with the odors ON (black) vs OFF (red). Right: Mean hyperpolarization (scaled as before) for the two cell groups over the two conditions. n = 8, 6 cells; P = 0.052, 0.97 for PV and SST; paired-sample two-sided t-test. **d**. ΔF/F traces from individual PV (top) and SST (bottom) cells and corresponding averages (thick lines), during the first odor cue, in trials with lowest (left, 5^th^ percentile) versus highest locomotion (right, 95^th^ percentile of average locomotion during odor). Corresponding locomotion traces shown in red. **e**. Left: Mean hyperpolarization over preferred vs non-preferred trials for PV and SST odor-specific field cells (P > 0.5, paired-sample two-sided t-test). Middle: Mean hyperpolarization in odor-specific, non-specific field cells and no-field cells. Right: frequency of hyperpolarization occurrence for the three cell groups (P > 0.05; two-sided WT). **f**. Hyperpolarization amplitude (top) and rate of occurrence (bottom) in PV cells in correct versus error trials (left, P > 0.5, paired-sample two-sided t-test) and as a function of performance (right) across trained sessions. **g**. Same for SST cells. **h**. Hyperpolarization amplitude (top) and rate of occurrence (bottom) in PV cells, as a function of locomotion during the hyperpolarization (left), the firing rate after the hyperpolarization (middle) and theta amplitude during the hyperpolarization (right). **i**. Same for SST cells. Black lines in **f-i**: Least squares estimate. ** P < 0.01, *** P < 0.001, otherwise P > 0.05; *F*-test.

The fact that hyperpolarization was not present in every cell and every trial suggests that it was not artifactual. To exclude a vibration artifact, we detached the odor/air alternating valve (the only moving component) from the recording rig, but the deflection was unaffected (Extended Data Fig. 4). To exclude any artifactual increase in fluorescence (as ΔF/F is inverted for negatively tuned ASAP3), we expressed the positively tuned ASAP4 GEVI^31^ in a set of mice (*n* = 5 SST-Cre mice). Noninverted ASAP4 ΔF/F traces exhibited a decrease in fluorescence, equivalent to that with ASAP3. Furthermore, the hyperpolarization was not triggered by locomotion, as it was present irrespective of the mouse moving during odor onset (Extended Data Fig. 4). It was not triggered by odor detection either, as it was present even when odors were turned off, and its amplitude was similar during preferred and nonpreferred odors in odor-specific field cells. Its amplitude and rate of occurrence across trials were also similar between odor-specific, non-odor-specific and no-field cells (Extended Data Fig. 4).

Of note, theta-bandpassed ΔF/F traces, averaged across trials, were larger around the hyperpolarization (Fig. 4b,d,k). This was not due to a transient increase in theta power by the hyperpolarization itself. In fact, the deflection resulted in a transient increase in delta power, whereas intracellular theta increased throughout the odor delivery, not just at its onset (Fig. 4m–n). Instead, the increase in average theta-bandpassed ΔF/F at odor onset indicated a transient theta phase resetting, resulting in reduced theta phase variance across trials at odor onset (Fig. 4l).

Across days, the hyperpolarization amplitude in PV, but not SST cells, was lower in trained sessions than in naive ones (Fig. 4o), whereas the subsequent depolarizations were similar (*P* > 0.05, WT). Notably, in both cell groups the rate of hyperpolarization occurrence decreased across days and thus was lower in trained than in naive sessions (Fig. 4p). These changes were not associated with DNMS performance as occurrence rates and amplitudes were similar in correct and error trials and uncorrelated with performance (Extended Data Fig. 4). Amplitudes were also uncorrelated with coincident locomotion or with post-hyperpolarization firing rates; however, occurrence rates were anticorrelated with locomotion (Extended Data Fig. 4), in accordance with the opposite progression of the two variables across days.

Collectively, these findings reveal a novel inhibitory input on PV and SST interneurons, triggered by the onset of odor delivery but dissociated from odors or locomotion, that transiently resets intracellular theta oscillations.

### Hyperpolarization is preceded by PV spiking and synchronizes rebound theta spiking

We next examined the spiking correlates of PV and SST interneurons to odor onset hyperpolarization.

**Fig. 5:**
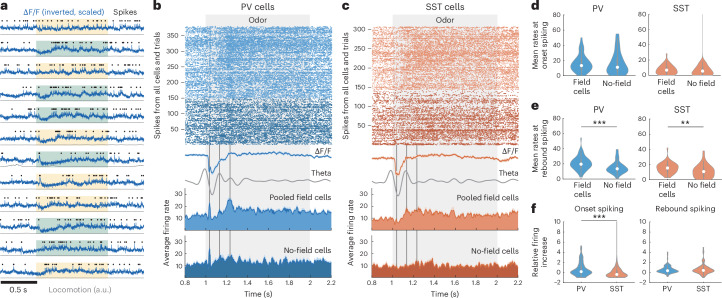
Brief synchronous spiking by PV cells, precedes PV and SST hyperpolarization and synchronizes rebound activity at theta peaks. **a**, Example trace of a PV cell around the first odor of a series of DNMS trials, displayed as before. Note the brief ‘onset spiking’, preceding the hyperpolarization in some, but not all, trials. **b**, Spikes of pooled PV field cells (light blue) and no-field cells (dark) around the first odor across all trials (top). Average finescale firing rates (binned at 5 ms) of the two cell groups aligned to the mean ΔF/F of all PV cells (blue trace) and its theta power-bandpassed signal (gray trace) (bottom). The three vertical lines are aligned to the first spike peak and the two theta peaks, respectively. **c**, Same for pooled SST cells. The three vertical lines indicate the same time points as in **b**. **d**, Mean firing rates at onset spiking (30–50 ms after odor onset) in field cells versus no-field cells (PV, 58 versus 49 cells; SST, 45 versus 48 cells). *P* = 0.403, 0.388; two-sided WT. **e**, Same for rebound spiking (200–500 ms after odor onset). *P* = 0.0004, 0.004; two-sided WT. **f**, Firing increase from baseline (0.5 s pre-odor) at onset and rebound spiking in PV versus SST cells (*n* = 107, 93 cells). *P* = 4.97 × 10^−4^, 0.89; two-sided WT. Single outlier truncated at **d** and **f** for plotting clarity.

PV cells often produced a single spike or short burst, immediately preceding a hyperpolarization (Fig. 5a), observed when pooling spikes across all cells and trials (Fig. 5b). In average PV firing rates with 5-ms binning (instead of 100 ms as before), this spiking yielded a peak at ~25 ms after odor onset, lasting ~30 ms and coinciding with the onset of average hyperpolarization. Notably, this brief odor onset spiking was largely absent in SST cells (Fig. 5c).

**Extended Data Fig. 5:**
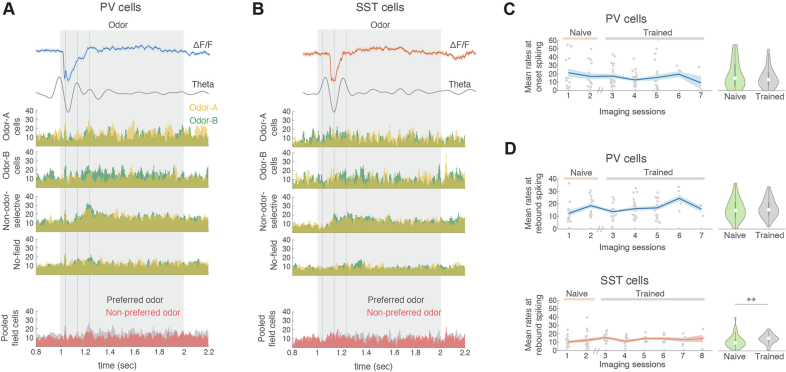
Fine-timescale spiking across different cell groups and odors, and its evolution across days. **a-b**. Average wideband and theta-bandpassed ΔF/F aligned with mean firing rates of (from top) odor A, odor-B, non-odor-specific and no field PV cells (**a**) and SST cells (**b**) across odor A (yellow) and odor-B (green) trials. Bottom: Rates of odor-specific cells in their preferred (gray) and non-preferred trials. **c**. Evolution of odor-onset spiking in PV cells (SST cells did not exhibit onset spiking) plotted as before. P = 0.54; two-sided WT. **d**. Evolution of rebound spiking in PV and SST cells. From top: P = 0.57, 0.0085; two-sided WT.

Moreover, the increase in odor spiking after the hyperpolarization was not uniform throughout the remaining odor interval. Instead, post-hyperpolarization rebound spiking was briefly modulated in theta cycles, corresponding to the reset intracellular theta peaks (Fig. 5b). The rebound spiking of SST cells was relatively attenuated, though theta modulation was still present in field cells (Fig. 5c). As average theta was synchronously reset in PV and SST cells (Fig. 4k), rebound spiking peaks were also synchronized between PV and SST cells (Fig. 5b,c). Similar features were observed between odor A and odor B trials and across preferred and nonpreferred odors of odor-specific cells (though, as expected, spiking was overall higher in preferred odors; Extended Data Fig. 5).

Odor-onset firing rates (30–50 ms into the cue) were similar between field and no-field cells (Fig. 5d) but rebound rates (200–500 ms) were higher in field cells compared to no-field cells (Fig. 5e). Even though PV cells fired more than SST during odor onset, the subsequent rebound firing increase was comparable between the two cell types (Fig. 5f).

Finally, PV odor-onset spiking did not change across days or pre- versus post-training; however, rebound spiking of SST cells, but not PV, slightly increased post-training (Extended Data Fig. 5).

Our findings reveal that the onset of hyperpolarization coincides with a brief synchronous PV spiking, whereas its offset is followed by rebound spiking of PV and SST cells, transiently synchronized in theta cycles.

### PV interneurons suppress most pyramidal cells during odors

After characterizing PV and SST interneuronal dynamics, we next sought to assess their effects on pyramidal activity during odor cues. We hypothesized that the prevalence of interneuronal odor fields should yield mostly inhibited pyramidal cells during the odor, rather than activated ones.

**Fig. 6:**
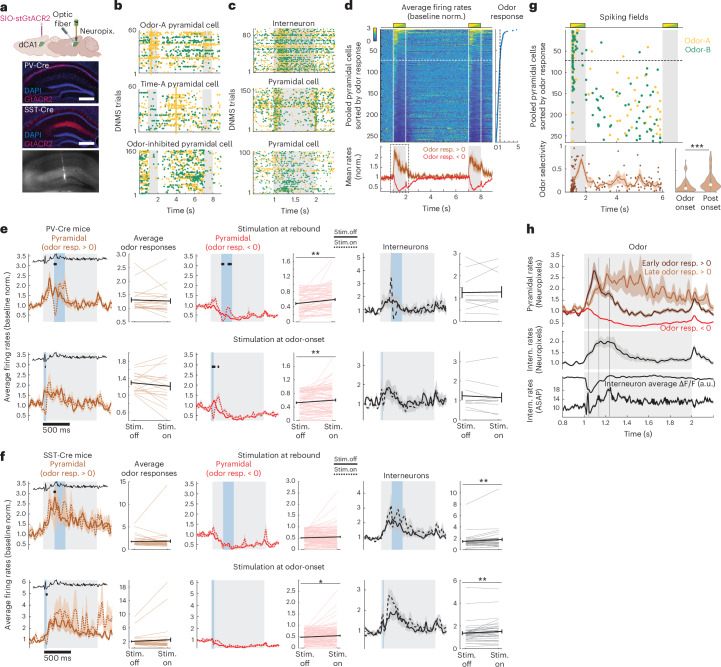
Inhibition shapes pyramidal odor responses. **a**, Schematic of surgical preparation for Neuropixel recordings with GtACR2 optogenetic stimulation (image created with BioRender). Example brain slices from a PV-Cre and an SST-Cre mouse showing GtACR2 expression in PV and SST cells, respectively. Scale bar, 50 µm. Slice showing Neuropixel probe placement (stained with CM-DiI) in CA1 (bottom). **b**,**c**, Raster plots of putative pyramidal units across DNMS trials (**b**) or interneuronal and pyramidal units during the first odor (**c**). Only trials without optogenetic stimulation shown. Spikes colored according to each trial’s first odor (yellow, odor A; green, odor B). Lines in **b** depict odor A-specific fields. **d**, Average firing rates, relative to baseline, of pyramidal units (*n* = 5 mice, 17 sessions), stacked by mean odor response during the first DNMS odor (right). Dashed lines separate cells with positive and negative odor responses. Mean ± s.e. rates across cells with positive and negative odor responses (bottom). **e**, Top row shows mean ± s.e. firing rates throughout the first DNMS odor of pyramidal units with positive and negative odor responses and interneuronal units from PV-Cre mice (*n* = 19, 77, 10 units) in trials without versus with optogenetic stimulation (solid and dashed lines) applied during the interneuronal rebound window (blue bars). Interneuronal firing rates from voltage imaging shown for reference (black). Black marks indicate time points with significantly different rates (*P* < 0.05, two-sided WT, FDR across the odor). Right-side panels show mean ± s.e. rates across the odor, without and with stimulation (from left, *P* = 0.64, 0.002, 0.81; paired-sample two-sided *t*-test). Bottom row shows same for stimulation during the odor-onset window (*n* = 17, 79, 10 units. From left, *P* = 0.28, 0.008, 0.3). **f**, Same as **e** for SST-Cre mice (top row, *n* = 36, 103, 35 units. From left, *P* = 0.78, 0.1, 0.005. Bottom row, *n* = 34, 105, 35 units. *P* = 0.191, 0.014, 0.008). **g**, Pyramidal odor A (yellow) and odor B (green) fields, sorted as in **d** (only cells with fields shown). Dashed line as in **d**. Odor selectivity of field cells (dots; small jitter added for clarity) and mean ± s.e. across cells (line) (bottom). Odor selectivity of early fields versus post-hyperpolarization fields (<200 ms and >200 ms from odor-onset, respectively; *n* = 56, 124 units) (right). *P* = 1.13 × 10^−5^, left-tailed *t*-test. **h**, Mean ± s.e. firing rate around the first odor (dashed box in **d**), from pyramidal units with negative odor responses (red) and with positive odor responses and peak rates within (dark brown) or after the hyperpolarization window (light brown; <200 ms and >200 ms from odor-onset, respectively) (top). Mean ± s.e. interneuronal unit firing rates (middle). Mean ΔF/F and mean ± s.e. firing rates of pooled interneurons from voltage imaging (bottom). Vertical lines show same time points as in Fig. 5b,c.

**Extended Data Fig. 6:**
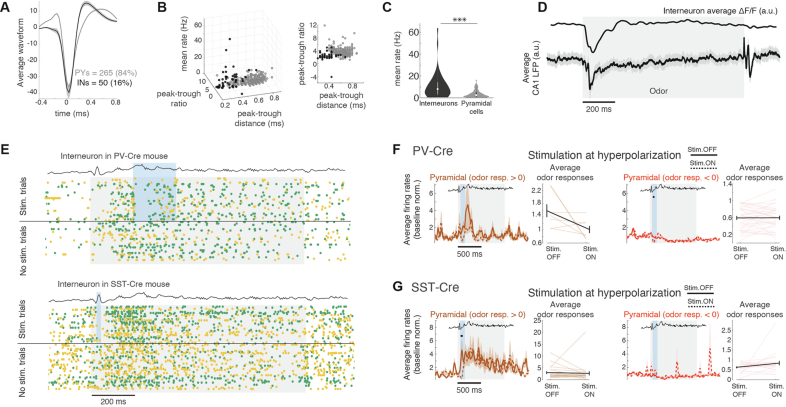
Electrophysiology and optogenetics during DNMS. **a**. Average action potential waveforms from all units identified as putative pyramidal cells and interneurons. **b**. Distribution of waveform peak-to-trough ratios versus peak-to-trough time distances versus mean firing rates for the two clusters. Right: Projection of peak-to-trough ratios versus distances. Dots: individual units, colored by cluster. **c**. Mean firing rates of putative pyramidal cells and interneurons (P = 7.3 × 10-9; two-sided WT). **d**. Average CA1 pyramidal layer LFP trace across the first odor in DNMS across mice. Top: Average ΔF/F from all interneurons recorded with voltage imaging. **e**. Top: Raster plot from putative interneuronal unit in a PV-Cre mouse, across DNMS trials without stimulation and with stimulation during the rebound window (blue bar). All trials shown were from same trial block (randomly mixed). Spikes colored by each trial’s first odor (yellow: odor A, green: odor-B). Superimposed interneuronal firing rates from voltage imaging shown for reference (black). Bottom: Same for interneuron from SST-Cre mouse and stimulation during odor-onset. **f**. Average firing rates of putative pyramidal cells from PV-Cre mice, during the first DNMS odor, with and without optogenetic inhibition of interneurons during the hyperpolarization window, plotted as in Fig. 6 (from left: P = 0.096, 0.956 for average odor responses; two-sided WT). **g**. Same for SST-Cre animals (from left: P = 0.69, 0.092 for average odor responses; two-sided WT).

To test this hypothesis, PV-Cre and SST-Cre mice were virally transfected, bilaterally in dorsal CA1, with the soma-targeted, Cre-dependent inhibitory opsin GtACR2 (ref. ^32^), which can efficiently silence neuronal firing without rebound spiking after light offset^33^. They were also implanted daily with Neuropixel probes and optic fibers above CA1 (either bilaterally or unilaterally; Fig. 6a). An additional wild-type mouse was only implanted with a Neuropixel probe unilaterally. We recorded extracellular spikes and local fields potential (LFP) signals during DNMS trials both in naive and trained sessions. Putative pyramidal and interneuronal units were clustered, based on spike waveforms and firing rates (Extended Data Fig. 6). Either PV or SST interneurons were optogenetically inhibited in randomly distributed subsets of trials (see below).

We first focused on pooled trials without optogenetic stimulation. Putative pyramidal units exhibited odor-specific fields during the odor (‘odor cells’) or delay period (‘time cells’), in accordance with the pyramidal sequences we have previously described^5^ (Fig. 6b). Moreover, putative interneuronal units increased spiking during the odor, in a non-odor-selective manner (Fig. 6c), as in our voltage imaging data. Of note, many pyramidal units reduced their activity or stopped spiking during the odor (Fig. 6b). Across pooled pyramidal units, only 26.4% increased spiking during odors (‘odor-excited’), whereas the remaining 73.6% had negative odor responses (‘odor-inhibited’, Fig. 6d), supporting our hypothesis that most pyramidal cells are inhibited during odor cues.

To test causally the role of inhibitory dynamics, observed with voltage imaging, on pyramidal odor responses, we optogenetically suppressed PV or SST interneurons, in PV-Cre and SST-Cre mice, respectively, during (1) the short window of PV spiking at odor-onset; (2) the hyperpolarization window; or (3) the rebound window. These stimulation protocols were delivered in separate trial blocks where they were randomly intermixed with nonstimulation trials (Methods). Many putative interneurons reduced spiking during stimulation (Extended Data Fig. 6), whereas others increased spiking, suggesting a release from recurrent inhibition.

Suppressing PV cells during odor-onset or the rebound window produced no effect in the firing rates of odor-excited pyramidal units (Fig. 6e; apart from a brief suppression at light onset due to transiently activated interneurons by the opsin^32^); however, it activated odor-inhibited pyramidal cells, significantly increasing their average odor responses (Fig. 6e). PV suppression had no significant effect on other interneurons.

Suppressing SST cells produced no effect in either pyramidal group, except for a small, significant increase in the spiking of odor-inhibited cells when stimulating at odor onset (Fig. 6f); however, it increased activity of other interneurons, resulting in significantly higher interneuronal odor responses (Fig. 6f), in agreement with findings that cortical SST cells inhibit other interneurons more strongly than PV cells do^34–36^.

Finally, no significant effects were observed when suppressing PV or SST cells during the hyperpolarization window, where their activity would already be minimal (Extended Data Fig. 6).

Collectively, our findings corroborate that PV-mediated inhibition effectively suppresses ‘background’ activity of most pyramidal cells during odors, allowing only a few pyramidal cells to respond to cues by spiking.

### Inhibition coordinates odor-cell and time-cell spiking

How is the spiking of the few odor-excited pyramidal cells temporally organized, relative to the interneuronal hyperpolarization and rebound? One possibility is that pyramidal cells also exhibit a hyperpolarization and they spike afterwards together with interneuronal rebound. Alternatively, the hyperpolarization may create a window for pyramidal spiking.

To address these alternatives, we first confirmed that putative interneuronal units exhibited a window of theta-modulated spiking, concurrent with the rebound window in our voltage imaging data (Fig. 6h). Out of the odor-excited pyramidal units, some fired during the hyperpolarization window without odor selectivity, whereas others fired mostly after the hyperpolarization with visible odor selectivity (Fig. 6c). As such, odor-excited pyramidal units could be split into two groups: those with peak activation before the rebound window (<200 ms from odor onset, ‘early-spiking’) and those with peak activation during or after it (>200 ms, ‘late-spiking’). In the early-spiking group, activity peaked explicitly during the interneuronal hyperpolarization window and quickly subsided, suggesting a brief disinhibition of these cells (Fig. 6h). Pyramidal excitation during the hyperpolarization window was also reflected in concurrent negative deflections in LFP traces (Extended Data Fig. 6). In the late-spiking group, activity was sparse during the hyperpolarization and increased together with interneuronal rebound (Fig. 6h). Therefore, inhibitory dynamics can split pyramidal odor responses into two subtypes.

Finally, we asked how odor and time cells relate to these pyramidal odor responses. Out of the pooled pyramidal units, sorted by their odor response (Fig. 6d), we isolated odor cells and time cells (Fig. 6g and Methods), collectively referred to as ‘sequence cells’. As expected, most odor cells belonged to the odor-excited group (60.5% of odor cells had positive average odor responses). The rest were mainly early-spiking cells with significantly lower odor selectivity (Fig. 6g) and reduced firing after the hyperpolarization window. Notably, most time cells belonged to the odor-inhibited group (93.6% of time cells) as opposed to increasing their firing during odors (but with a lower peak than during their delay time field). In accordance with these findings, optogenetic suppression of PV or SST interneurons had no effects on odor cells, as these belonged to the odor-excited group and were thus unaffected by stimulation (*P* > 0.05, paired-sample *t*-test of average odor responses in all stimulation protocols). Accordingly, PV or SST suppression during odor onset and PV suppression during rebound significantly increased odor spiking of time cells (*P* < 0.05 and *P* < 0.01, respectively) as these belonged to the odor-inhibited pyramidal group.

**Extended Data Fig. 7:**
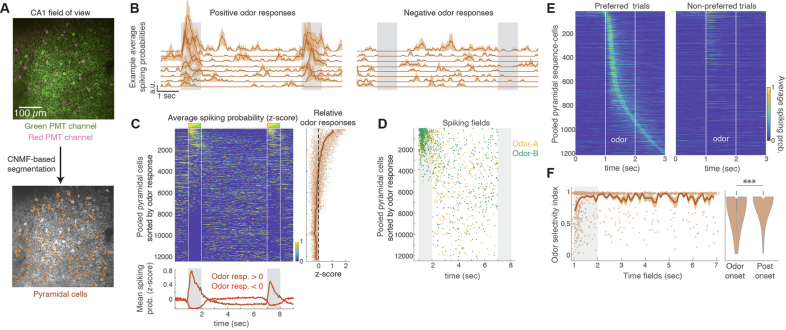
Calcium imaging-based pyramidal odor responses are shaped by inhibition. **a**. Example field of view from two-photon calcium imaging in CA1 pyramidal layer of a Gad2-Cre:Ai9 mouse expressing GCaMP6f (green) in all cells and tdTomato in all GABAergic cells (magenta). Right: Same FOV after ROI segmentation of pyramidal cells. **b**. Example traces of average deconvolved signal (‘spiking’) across all trials in cells with positive (left) and negative odor responses (right). **c**. Average z-scored spiking of pooled cells (N = 11 mice, 58 sessions), stacked by their mean response during the first odor. Note that many cells stop spiking during the odor. Right: z-scored odor responses averaged across the odor duration. Most cells exhibit a negative response. Bottom: Mean spiking across all cells with positive (brown) and negative (red) odor responses. **d**. Field time bins of pooled Odor A (yellow) and Odor-B (green) sequence-cells, sorted according to the order in C. Most time cells (70.8%) have negative odor responses. **e**. Average spiking (scaled) of pooled pyramidal sequence-cells, sorted by time field. Spiking is zoomed around the first odor in preferred (left) and non-preferred trials (right). Cells with fields after this time window are omitted. Note the response in non-preferred trials of early odor cells. **f**. Distribution of odor selectivity index from all sequence-cells (dots; small jitter added for plotting clarity) and average (thick trace). Multiple early odor cells have low selectivity index. Right: Selectivity index of early odor cells (field < 250 msec post odor-onset) vs the remaining sequence-cells (P = 1.82 × 10^−18^, left-tailed WT).

To ensure our observations generalize to a larger sample of sequence cells from the defined CA1 pyramidal layer, we analyzed a large dataset from two-photon calcium imaging in dorsal CA1 of transgenic mice (*n* = 11), virally transfected with GCaMP6f, during the DNMS task^5^ (Extended Data Fig. 7). A total of 12,476 identified pyramidal cells were pooled across 58 imaging sessions. Odor-specific pyramidal spiking sequences were previously observed in these recordings^5^. Using the deconvolved calcium signal as a proxy of spiking probability, we confirmed that (1) the majority of pyramidal cells were inhibited by the odor (67.3% of pyramidal cells); (2) odor cells with a field during the interneuronal hyperpolarization window had lower odor selectivity than those spiking post-hyperpolarization; and (3) most time cells had a negative odor response (70.8% of time cells; Extended Data Fig. 7).

These results suggest that time cells are largely inhibited during odor cues, whereas odor cell responses can be segmented based on inhibitory spiking dynamics, with odor-selective cells spiking together with interneuronal rebound.

## Discussion

Using voltage imaging in vivo, we investigated how interneurons interact with hippocampal pyramidal sequences that encode and retain sensory or contextual information across gaps in time^1,2^. Unlike odor-specific sequences throughout cues and the ensuing delay in a working memory task, CA1 PV and SST interneurons encoded odor delivery but not odor identity or delay time. Odor-triggered inhibition was exerted by a stable number but different set of cells daily, irrespective of whether the animal was trained to the task or not. Transient PV spiking at odor onset was followed by a widespread hyperpolarization, which reset intracellular theta and synchronized rebound PV and SST spiking. Electrophysiological recordings, two-photon calcium imaging and optogenetic manipulations corroborated that PV interneurons silenced most pyramidal cells during odor cues, including time cells, whereas SST interneurons suppressed mostly other interneurons. The few odor-activated pyramidal cells spiked in coordination with interneurons. Our collected findings suggest that inhibition mediates memory-encoding (cue presentation) rather than maintenance (delay period), by increasing the signal-to-noise ratio of pyramidal cue representations. This may be crucial for efficiently encoding sensory information and conveying it downstream.

So far, most insights on the role of inhibition in hippocampal dynamics in vivo have come from navigation tasks revealing diverse inhibitory modalities, including spatially tuned^37^, untuned^38^ and negatively tuned inhibition^39,40^. Whether and how interneurons mediate nonspatial, sensory and temporal representations by pyramidal cells remained underexplored. Our findings combine temporally tuned inhibition during cues, untuned inhibition during the delay period, and no systematic negatively tuned fields, suggesting that inhibitory tuning depends on the behavioral context.

Our results indicate that interneurons regulate working memory-related pyramidal dynamics in a few ways. First, interneurons increase the signal-to-noise ratio of sensory representations by suppressing background through their widespread, nonselective activation, so that only a minority of pyramidal cells can spike in response to a cue. Our finding that this function is mainly performed by PV interneurons agrees with the efficient suppression of action potentials by PV interneurons^41^. A similar role has been reported during navigation, where spatially homogeneous inhibition suppressed out-of-field excitation and enhanced the selectivity of place cells within their fields^38^. In the cortex, a similar role in sharpening pyramidal stimulus selectivity or spike timing has been proposed^42^. Of note, interneuronal ensembles performing this function exhibited fast cell turnover across days, contrasting the slow drift of odor-encoding pyramidal cells^5^ and arguing against a hard-wired class of inhibitory neurons for regulating hippocampal odor encoding.

Another inhibitory effect on cue representations may be the segmentation of pyramidal responses into early non-odor-selective ones and late odor-selective ones, coinciding with theta-paced interneuronal rebound. This segmentation could be driven by a different degree of PV-mediated inhibition that CA1 pyramidal cells receive. PV interneurons are known to synchronize rebound pyramidal theta spiking in vitro^43^ and in vivo^17^ and a similar theta resetting was observed in rat CA1 EEGs during odors, ~150 ms after sniffing onset^4^, as the timeline reported here. In the same study, odor-responsive pyramidal cells increased spiking ~300 ms after sniffing onset, like the late-spiking group here. Theta-organized responses can better entrain downstream cortical targets^44^, efficiently transmitting cue information across hippocampal–cortical circuits.

The source of the interneuronal hyperpolarization remains unclear. It may be triggered by the brief PV spiking at odor onset, whose temporal precision suggests an external drive, rather than recurrent inputs. Its ~20 ms lag after cue-onset, though consistent with the latency between olfactory bulb (OB) stimulation and hippocampal response in rats^45,46^, would discount the diffusion time needed for the odorant to reach the epithelial cells and then the OB, or any sniffing cycle variability across trials. This synchronous spiking should also hyperpolarize both interneurons and pyramidal cells. Alternatively, the hyperpolarization may be driven by a common theta-paced GABAergic input from the medial septum on CA1 PV and SST interneurons^47,48^, which could explain the high interneuronal synchrony as well as the theta-modulated rebound. The hyperpolarization may also be triggered by interneuron-specific VIP cells^49^ recruited by lateral entorhinal axons^50^. VIP-mediated disinhibition could explain the pyramidal spiking during the hyperpolarization.

The behavioral correlates of the hyperpolarization are also unclear. Low odor selectivity of early-spiking pyramidal cells implies that responses <200 ms from odor onset may be driven by inputs unrelated to olfaction, such as the clicking sound of the odor-delivering air valve or a startle response to it. Indeed, mouse CA1 pyramidal cells can exhibit a tone-induced or startle-induced hyperpolarization after ~120 ms (ref. ^51^). The entorhinal cortex also responds to auditory cues, through glutamatergic medial septal inputs, within <15 ms (ref. ^52^) and could drive early-spiking cells in CA1. On the other hand, the absence of a hyperpolarization in every trial and the reduction of its occurrence across days, argue against a purely auditory response and in favor of a startle or arousal response.

A striking difference with pyramidal sequences, is the sparsity of interneuronal fields during the delay, suggesting that PV and SST cells receive different inputs than time cells. As most SST cells are driven by feedback CA1 excitation, they may integrate inputs from multiple time cells during the delay, yielding no temporal tuning. Similarly, PV cells may integrate various temporally tuned inputs from CA3 and EC^53^, both of which form spiking sequences similar to CA1 (refs. ^54–56^).

One limitation in our approach is the lack of differentiation between diverse subtypes of PV or SST interneurons with distinct morphology and functional roles^15,23^ and different relationships to network patterns^25^. Imaging single cells in a small FOV also misses important population interactions. Future experiments with transgenic lines allowing more cell-type-specific GEVI expression, as well as recordings with different GEVIs expressed on different cell types simultaneously^57^, will disentangle the role of specific interneuronal subtypes and their rich interplay.

However, our findings highlight how voltage imaging can reveal cell-type-specific spiking and membrane potential dynamics during active behavior, with high temporal resolution^58,59^ and across long time scales of several days. Recent developments of GEVIs with improved fluorescence, kinetics and photostability^28,31,57,60^, render voltage imaging ideal for long-term recording membrane potentials from genetically identified cells in vivo.

## Methods

### Animals

A total of ten adult male mice (12–21 weeks old) were used for in vivo voltage imaging experiments with the ASAP3 GEVI: five PV-Cre mice (B6;129P2-*Pvalb*^*tm1(cre)Arbr*^/J) and five SST-IRES-Cre mice (*Sst*^*tm2.1(cre)Zjh*^/J). For ASAP4 recordings, five adult SST-IRES-Cre adult mice were used.

For electrophysiological recordings, three adult male and two female mice (8–16 weeks old) were used: two PV-Cre, two SST-Cre and one wild-type (*Sst*^*tm2.1(cre)Zjh*^/J^−/−^).

For calcium imaging recordings, 11 adult mice (11–31 weeks old) were used: 5 Gad2-Cre:Ai9 mice (Gad2^tm2(cre)Zjh^/J crossed with B6.Cg-Gt(ROSA)26Sor^tm9(CAG−tdTomato)Hze^/J) and 6 Gad2-Cre:Ai14 (Gad2^tm2(cre)Zjh^/J crossed with B6.Cg-Gt(ROSA)26Sor^tm14(CAG−tdTomato)Hze^/J).

All animals were acquired from the Jackson Laboratory and were group housed (2–5 per cage) on a 12-h light–dark cycle. All experimental protocols were approved by the Chancellor’s Animal Research Committee of the University of California, Los Angeles, in accordance with the National Institutes of Health (NIH) guidelines.

### Surgical procedures

Surgical procedures were described in detail in ref. ^5^. In brief, isoflurane-anesthetized mice were stereotactically injected with 500 nl undiluted AAV8-ef1α-DiO-ASAP3-Kv (titer 3.83 × 10^12^ µg ml^−1^), using a Nanoject II microinjector (Drummond Scientific) into the right dorsal CA1 area (−2mm posterior and 1.8 mm lateral to bregma, 1.3 mm ventral from dura) at 60 nl min^−1^. After 1 h, a circular craniotomy (3-mm diameter) was made around the injection site and the cortical tissue above the dorsal CA1 was aspirated until partial removal of the corpus callosum. A 3-mm titanium ring with a glass coverslip attached to its bottom was implanted into the aspirated area and secured to the skull. A custom-made lightweight metal head holder (headbar) was attached to the skull posterior to the implant. Cyanoacrylate glue and black dental cement (Ortho-Jet, Lang Dental) were used to seal and cover the exposed skull. Mice were administered carprofen (5 mg kg^−1^ of body weight) for 3 days post-surgery, as well as amoxicillin antibiotic (0.25 mg ml^−1^ in drinking water) through the water supply for 5 days.

For ASAP4 recordings, the same procedure was followed but two animals were injected with AAV8-ef1α -DiO-ASAP4b-Kv (titer 2.36 × 10^12^ vg ml^−1^) and three animals were injected with AAV8-ef1α-DiO-ASAP4e-Kv (titer 3.45 × 10^11^ vg ml^−1^). All ASAP viruses were produced by the Stanford Neuroscience Gene Vector and Virus Core facility.

For two-photon calcium imaging, the same procedure was followed but animals were injected with 1,500 nl of 1:10 saline-diluted AAV1.Syn.GCaMP6f.WPRE.SV40 virus (diluted immediately before surgery; titer 4.65 × 10^13^ GC ml^−1^; Penn Vector Core).

For Neuropixel recordings, mice were subcutaneously administered preoperative drugs (carprofen 5 mg kg^−1^, dexamethasone 0.2 mg kg^−1^, lidocaine 5 mg kg^−1^) 30 min before surgery, were then isoflurane-anesthetized and were placed into a stereotactic frame, as before. After stereotactically aligning the skull, burr holes were made and AAV1_hSyn1-SIO-stGtACR2-FusionRed (1,000 nl of 1:10 saline dilution; Addgene) was injected bilaterally (−2.0 mm posterior, 1.8 mm lateral, 1.3 mm ventral from dura) in all mice except the wild-type. A headbar was attached to posterior skull and secured with cyanoacrylate glue and dental cement as before. The remaining exposed skull and brain were covered and sealed with a silicone elastomer sealant (Kwik-Sil, WPI). Following surgery, all animals were given postoperative care (carprofen 5 mg kg^−1^ and dexamethasone 0.2 mg kg^−1^ for 48 h after surgery) and provided amoxicillin-treated water at 0.5 mg ml^−1^ for 7 days. After waiting for 3 weeks to allow viral expression, an additional surgery was performed for all mice. Additional small burr holes were made 1.5 mm anterior to viral injections for acute cannula placement. Another small burr hole was made above the right cerebellum, and a silver chloride ground wire was implanted within the hole and fixed in place with dental cement. The exposed skull and brain were again covered and sealed with the silicone elastomer sealant (Kwik-Sil, WPI). Following surgery, all animals were given postoperative care (carprofen 5 mg kg^−1^ and dexamethasone 0.2 mg kg^−1^ for 48 h after surgery) and provided with amoxicillin-treated water at 0.5 mg ml^−1^ for 7 days.

### Experimental setup for imaging

The behavioral rig used for DNMS recordings was described in ref. ^5^. In brief, mice were head-fixed through a custom-made headbar holder on an 8-inch Styrofoam ball treadmill rotating in one dimension. Locomotion was recorded using a custom-printed circuit board based on a high sensitivity gaming mouse sensor (Avago ADNS-9500), connected to a microcontroller (Atmel Atmega328). A constant stream of clear air (~1 l min^−1^) was supplied to the mouse through a custom-made lickport. During odor stimulation a dual synchronous three-way valve (NResearch), switched from the clear air stream to the odorized air for 1 s. Odorized air was created using a four-port olfactometer (Rev. 7c; Biology Electronics, Caltech), supplying air to either of two glass vials containing liquid isoamyl acetate (70% isoamyl acetate basis, FCC; Sigma-Aldrich) or pinene ((−)-α-pinene, ≥97%, FCC; Sigma-Aldrich) odorants, diluted in mineral oil at a 5% concentration. Odorized air reached the rig through Tygon tubing, leading to a dual three-way solenoid valve (Lee Company). The olfactometer supplied air to either odor vial 1 s before actual stimulation to allow for odorized air to travel through the tubing. During the 1-s stimulation, a three-way solenoid released it to the mouse through the lickport. At the offset of the stimulus, the valve switched the airstream back to clear air, ensuring a constant flow of air to the mouse and a quick clearing of the odorant from the air around the mouse. The odorized and clean air was set to similar airflow values (~1 l min^−1^), measured with a flowmeter (AWM3300; Honeywell). Licking was detected using a battery-operated, custom-made, printed circuit board operating as a lickometer. Water droplets (~10 µl) were released by a solenoid valve (Lee Company). At the end of each trial, after the response window, vacuum was applied for 3 s to clear any lingering water and remove any odorized air. An additional 7 s of intertrial interval were applied after the vacuum. The behavioral rig was controlled with custom-written software (MATLAB) and through a data-acquisition board (USB-6341; National Instruments).

Voltage imaging was performed with a custom-built, high-speed, single-photon, epifluorescent microscope. Photoexcitation was provided via a fiber-coupled LED (Thorlabs, M455F3) with a center wavelength of 455 nm. Excitation light was collimated after a 2-m long, 400-μm core multimode fiber optic patch cord (Thorlabs, M28L02) and expanded using a Keplerian telescope. The expanded beam was passed through a spectral excitation filter (Thorlabs, MF455-45) and reflected off a long-pass dichroic mirror (Thorlabs, MD480) before reaching a ×16/0.8 NA water-immersion objective (Nikon, CFI75 LWD ×16 W). The expander was used to generate a localized excitatory spot ~165 μm in diameter, at the focal plane. Emitted fluorescence was collected and transmitted through the dichroic mirror and an emission filter (Thorlabs, MF530-43) before reaching a 100-mm tube lens (Thorlabs, AC300-100-A) to form an image on a fast scientific CMOS camera (Hamamatsu Photonics, ORCA-Lightning C12120-20P) capable of kHz frame rates. The spatial sampling rate of the microscope was calculated as ~688 nm and experimentally confirmed using a calibration test target. Camera control and image acquisition were performed using HCImage Live software v.4.5.1 (Hamamatsu Photonics).

Calcium imaging was described in ref. ^5^. In brief, a resonant scanning two-photon microscope (Scientifica) was used, recording 512 × 512 pixel frames at 30.9 Hz, with the same objective as for voltage imaging (500 × 500 µm FOV). Excitation light was delivered with a Ti:sapphire excitation laser (Chameleon Ultra II, Coherent), operated at 920 nm. GCamp6f and tdTomato fluorescence were recorded with green- and red-channel gallium arsenide photomultiplier tubes, respectively (Hamamatsu). Microscope control and image acquisition were performed using LabView-based software (SciScan).

For all recordings, imaging and behavioral data were synchronized by recording voltage pulses, generated at the onset of each imaging frame, as well as olfactory stimulation digital signals at 1 kHz, using the WinEDR software (Strathclyde Electrophysiology Software).

### Experimental setup for electrophysiology

Following at least 24 h of recovery after the final surgery, recordings took place during DNMS trials, using the same behavioral rig as before. Mice underwent two recording sessions while naive to DNMS. Following the second recording session, mice began water deprivation and received DNMS training. Recordings resumed 10–11 days later. At each session, the elastomer sealant was removed and mineral oil was immediately placed on top of the craniotomy to protect the exposed brain. The mouse skull was stereotactically aligned. Neuropixels 1.0 probes (Imec) were lowered, either unilaterally or bilaterally, using a micromanipulator into the hippocampus for 2.8 mm and were allowed to settle for at least 10 min. Recordings were performed at 30 kHz sampling rate, using PCI eXtensions for Instrumentation (PXI) hardware from National Instruments and SpikeGLX data-acquisition software (http://billkarsh.github.io/SpikeGLX/). During some recordings (once per mouse), Neuropixels were coated with a fluorescent dye (CM-Dil, Sigma-Aldrich) before lowering.

For optogenetic experiments in the PV-Cre and SST-Cre mice, optic fiber cannulas (400 µm Core, 0.39 NA, Thorlabs) were placed 1.5 mm anterior to the Neuropixel insertion (unilaterally or bilaterally accordingly) and lowered for 2 mm at 45°, so that fiber tips sat ~0.1 mm anterior of the probe and at ~1.4 mm depth. Light stimulation was delivered from a 470 nm fiber-coupled LED (Thorlabs) with power set at 4 mW out of the optic fiber. Three different stimulation protocols were used: (1) stimulation started 30 ms after odor onset and lasted 20 ms, covering the brief PV synchronized spiking at odor onset; (2) stimulation started 50 ms after odor onset and lasted 100 ms, covering the mean hyperpolarization window; and (3) stimulation started 200 ms after odor onset and lasted 200 ms, covering the mean post-hyperpolarization rebound window. The same stimulation was delivered during both odors in each DNMS trial. In each recording session, after a set of trials without any stimulation, the protocol was alternated after one or more blocks of 20 trials (maximum four blocks before changing protocol). Protocols 1 and 3 were delivered for at least one trial block in every session. Protocol 2 was delivered during four trained sessions (covering all three mice). Stimulation was given in a random set of trials (50% on average). In a final set of trials, stimulation was given throughout the entire odor cues in each trial, but this only suppressed interneuronal spiking for the first 100–200 ms (possibly due to inhibitory compensation mechanisms) and had minimal effects on pyramidal cells. In three sessions, a block of no-stimulation trials was also recorded at the end.

Following the completion of each recording, Neuropixels and fiber cannulas were stereotactically removed. The exposed brain and skull were rinsed with cortex buffer (135 mM NaCl, 5 mM KCl, 5 mM HEPES, 1.8 mM CaCl_2_ and 1 mM MgCl_2_) and the elastomer sealant Kwik-Sil was replaced.

### Histology

Following all experiments, mice were deeply anesthetized under isoflurane and transcardially perfused with 30 ml 1× PBS followed by 30 ml 4% paraformaldehyde in 1× PBS at a rate of approximately 4 ml min^−1^. After perfusion, the brains were extracted and post-fixed in 4% paraformaldehyde. Coronal sections of 80 μm were collected using a vibratome, 24–48 h after perfusion and were mounted onto glass slides and cover-slipped with 4,6-diamidino-2-phenylindole (DAPI) mounting medium. Images were acquired on an Apotome2 microscope (Zeiss; 679 ×5, ×10 and ×20 objectives) to confirm proper expression and location of viral SIO-stGtACR2 expression, as well as stained Neuropixel probe depth. Sufficient expression in PV or SST interneurons localized within CA1, in sections from PV-Cre or SST-Cre mice accordingly, and proper probe placement were confirmed.

### Behavioral training and voltage imaging protocol

Approximately, 2–4 weeks after surgery, mice were imaged for two sessions (separate days) while completely untrained in the DNMS task (one SST-Cre mouse was imaged for three sessions). They were presented with sets of DNMS trials and could passively smell the odor cues. Locomotion on the treadmill was typically limited as mice were not accustomed to the rig. After the second session, water restriction was initiated and DNMS training began in separate training rigs. Protocols for water restriction and training to the DNMS task, starting ~10 days after surgery, were described in ref. ^5^. After mice reached performances >90% for at least one 20-trial set (75% for one animal; one animal never reached criterion but only two cells were recorded post-training), voltage imaging resumed. The recording protocol was the same for naive and trained performance (see below). Daily recordings typically stopped when either the mouse had stopped performing due to water satiation or no more cells with sufficient signal could be detected.

All imaging was performed under the custom-made single-photon microscope at 1,000 frames per second. Before each recording, we searched for cells with strong epifluorescence through a 352 × 352 µm FOV (256 × 256 pixels and 2 × 2 pixel-binning). Once a cell was located, the FOV was reduced to 88 × 44 µm (64 × 32 pixels with 2 × 2 pixel-binning), which allowed for longer camera exposure (0.98 ms) and digitization at a 1-kHz frame rate. The FOV contained a single cell in most cases with only three PV cell recordings (2.8% of all sessions) and two SST recordings (2.2%) containing >1 cells within the FOV with all cells yielding action potentials. Before DNMS recordings, 3 s of activity were recorded. Using ImageJ, we manually segmented the recorded video and examined the raw fluorescence of the ROI. Only cells that yielded clear action potentials within the 3-s videos were further recorded. The power of the LED was adjusted depending on the signal-to-noise ratio in the 3-s recording. LED power was typically set as low as possible to avoid fast photobleaching but also to allow large spike amplitudes. The number of DNMS trials to be delivered was also set based on an assessment of the signal and the rate of photobleaching.

At each DNMS trial, LED and frame acquisition were on from 1 s before the first odor onset until the offset of the response window (11 s total). LED and frame acquisition were off during the 10 s total intertrial interval. After a set of trials was recorded, a time break of ~5–10 min was given to prevent phototoxicity, while the mouse remained head-fixed under the microscope. During this break, the acquired raw traces were examined, using ImageJ, for the existence of observable action potentials. If action potentials persisted throughout the final trial, a new recording of the cell was initiated over an additional set of trials. Otherwise, the cell was considered photobleached. In most cases, recording of a cell stopped after an adequate number of trials was recorded, before the cell was bleached. This allowed us to record cells for multiple consecutive days. After recording a particular cell, the FOV was expanded back to 352 × 352 µm and a small number of frames were recorded. The average frame of this video (ImageJ) was used as reference to locate the same cell on following days, based on cell body shape as well as processes and surrounding features.

For experiments with odors turned off, the olfactometer ports were manually deactivated in every other trial, halting any flow of odorized air. Trials were otherwise normal, including all auditory cues from the three-way valve. For detached air valve experiments, the three-way valve was manually detached from the rig for a subset of trials but remained connected and operational.

### Voltage imaging data processing

Each recorded video, together with the corresponding behavioral data, was processed separately using the Volpy automated pipeline for voltage imaging in Python^30^. The video was first motion corrected using the rigid NoRMCorre^61^ algorithm as implemented in the CaImAn toolbox^62^. Parameters like the size of a kernel for high-pass spatial filtering or the maximum rigid shift were adjusted between videos to achieve efficient motion correction. Initial ROI bounds were manually drawn around the recorded cell(s) using a graphical user interface in Volpy. Trace extraction, background removal and denoising together with spike time estimation took place through a reiterative process, based on the constrained nonnegative matrix factorization (CNMF) algorithm^63^ and a modified version of the SpikePursuit algorithm^64^. In each iteration, estimated background is removed from the extracted trace, spikes are selected from local maxima using an adaptive threshold, a spike template is computed and is used for whitened matched filtering the trace to enhance the spikes and then re-detect them. A reconstructed trace, obtained by convolving the spike template and the inferred spike train, is then used to refine the spatial footprint of the ROI through Ridge regression, and start the next iteration (typically three iterations performed). Final ΔF/F was computed as (*t* − *t*_sub_) / *t*_sub_ where *t* is the refined temporal fluorescence trace and *t*_sub_ is an estimated subthreshold trace. ΔF/F was inverted for all ASAP3 recordings but not for ASAP4 recordings. Parameters involved in trace denoising and spike detection, such as frequency for high-pass filtering to remove photobleaching or the maximum number of spikes to form the spike template, were adjusted between videos to ensure the accurate detection of action potentials that could be seen in the raw trace. Processing took place over the entire video with concatenated trials; traces and spike times were then split into the corresponding trials.

### Analysis of voltage imaging data

All analysis was performed using custom-written software in MATLAB.

#### Motion analysis

The locomotion signal was recorded at 1 kHz. Its mode value was subtracted, then it was turned to absolute values and smoothed with a first-order Savitzky–Golay filter. Motion segments were taken to be those where the locomotion signal exceeded 0.02 and immobility was assumed when it dropped 0.01 (a.u.). Consecutive motion segments closer than 20 ms were concatenated and segments shorter than 10 ms were discarded.

Immobility during the first odor (Extended Data Fig. 2), was considered when smoothed locomotion (50 ms moving average), averaged across the odor delivery, was <0.02 (a.u.). Otherwise, the trial was considered to have locomotion during the odor.

#### Spiking and subthreshold membrane potential analysis

Initially, all processed sets of trials from each neuron in a session, along with the behavioral data, were concatenated and considered one continuous recording of that cell. The quality of processing from each recording was assessed visually. Cells that yielded signals that were too noisy, with problematic spike detection or with no spikes, were removed from analysis. Trials at the end of a recording containing very few or no spikes, due to phototoxicity or bleaching, were also removed.

Firing rates were computed by binning the extracted spike times of each trial at 100 ms or 5 ms (Fig. 5 and Extended Data Fig. 5) nonoverlapping bins. Interspike intervals were computed for each trial separately. The burst index of a neuron was quantified as the percentage of spikes closer than 10 ms, over its total number of spikes. Spike amplitudes were computed as the ΔF/F difference between average spike peak and baseline (average ΔF/F from 20 to 60 ms before the spike) and halfwidths as the average distance between half-peak time points per spike.

Speed scores were computed as the trial-averaged correlations between a cell’s firing rates (smoothed with a five-point moving-average window in each trial) and the animal’s locomotion in the corresponding trials (downsampled to match the firing rate sampling rate). Odor scores were the trial-averaged correlations between a cell’s firing rate and a vector of trial length, containing 1 s in the odor delivery time bins and 0 s in all other time bins.

Analyses of subthreshold membrane potentials were based on de-spiked versions of the ΔF/F signal. Spike removal was performed through a Bayesian method, developed for removing spike waveforms from LFPs to eliminate artifactual spike–LFP correlations^65^. A segment of 25 ms around each detected spike (5 ms before and 19 ms after the action potential peak) was replaced in the ΔF/F based on Bayesian inference. Due to the action potential widening by GEVIs, this segment encompassed the average recorded action potential and the ensuing after-hyperpolarization.

Power spectra were computed separately on concatenated motion or immobility de-spiked ΔF/F segments from each cell, through a modified periodogram using a Kaiser window with a frequency resolution of 0.5 Hz. Power spectrograms were computed through Short-Time Fourier Transform of the de-spiked ΔF/F of each cell in each trial, across 512-ms windows with 256-ms overlap and 0.1 Hz frequency resolution. They were two-dimensionally interpolated and then smoothed with a two-dimensional Gaussian smoothing kernel (*σ* = 5). Mean spectrograms were fitted by a power law model that was then removed to flatten the spectrograms.

Theta phases and amplitudes were computed from the Hilbert transform of the de-spiked ΔF/F after bandpassing it in the 4–10 Hz frequency range, using a third-order Butterworth filter. The phase of each spike was computed as the angle of the Hilbert transform at the spike peak. Theta amplitude traces were smoothed with a first-order Savitzky–Golay filter. Mean amplitudes were estimated as the mean amplitude across all trials and all time points. Average theta phase of a cell’s spikes and mean vector length (strength of theta modulation) and phase variance were computed using the Circstat toolbox^66^. To compute the relationship between firing rates and intracellular theta power with locomotion or odors, (Extended Data Fig. 1), the first and last 700 ms in each trial were removed to avoid edge artifacts arising from small ΔF/F distortions when high-pass filtering the original, concatenated fluorescence trace to remove low frequency photobleaching components.

For hyperpolarization analysis, non-de-spiked ΔF/F traces were smoothed by first-order Savitzky–Golay filter and *z*-scored to the mean and s.d. of the 0.5 s preceding the onset of the first odor (baseline) in each trial separately. Time segments where this *z*-scored trace *S* was *S* < −1 during to the first odor delivery were detected. The first continuous time segment *T*_h_ (including all segments closer than 20 ms) was considered a potential hyperpolarization. If *T*_h_ had total duration of >50 ms and contained time points where *S* < −3 with total duration >10 ms, then *T*_h_ was considered a significant hyperpolarization. The first time point, the total length and the *S* minimum value of *T*_h_ were taken as the hyperpolarization onset time, duration and amplitude respectively. The timing of minimum *S* value was taken as the hyperpolarization trough timing. For comparisons including low number of recorded cells (Extended Data Fig. 5), the minimum value of *S* within the first 200 ms after odor onset, in every trial, was taken as the hyperpolarization amplitude.

For relationships between hyperpolarization amplitude and locomotion/theta/firing rates (Extended Data Fig. 4), average locomotion or theta amplitude was computed between each trial’s hyperpolarization onset and offset time points, in trials with significant hyperpolarization. Post-hyperpolarization firing rates were computed from hyperpolarization offset till end of odor cue. For relationships between rate of hyperpolarization and the above measures, average locomotion or theta amplitude was computed over the first 150 ms after odor onset and averaged across all trials, whereas post-hyperpolarization rates were computed after the first 150 ms till end of odor cue. Trials with the lowest and highest locomotion during hyperpolarization (Extended Data Fig. 4) were those where the average locomotion during hyperpolarization, in trials with significant hyperpolarization, were in the lowest or highest fifth percentile of the distribution, respectively.

For fine-timescale analysis (Fig. 5 and Extended Data Fig. 5), firing rates (5-ms bins) were smoothed with a three-point moving-average window. Odor-onset and rebound spiking corresponded to the bins within 30–50 ms and 200–500 ms after odor onset, respectively. Corresponding average rates referred to the mean firing rate value across the respective time bins and across all trials. Firing increase was computed as the relative change of the trial-averaged rate (mean across the corresponding time bins) to its baseline average rate (mean over the 0.5 s preceding the odor).

All plotted ΔF/F traces were normalized by their maximum value over each trial. Plotted firing rates over individual trials, as well as average rates, were *z*-scored based on their mean and s.d. across the odor delivery interval. Firing rates or theta amplitude traces were smoothed by a five-point moving-average window for plotting.

#### Field detection and analysis

The firing rate across each trial was smoothed with a five-point moving-average window. Firing rate signals over the odor-delay interval (from onset of first odor to end of the delay period) were *z*-scored in each trial and split over trials initiated by odor A or odor B. The average rate was computed for each set and the corresponding maximum values were noted for each. The two sets of firing rate traces were then circularly shifted by a random interval up to ± 1/2 odor-delay interval duration, separately for each trial, and the maximum average firing rate over the shifted trials was again computed. This process was repeated 1,000 times, generating a distribution of maximal rate values. The cell was considered to have a significant firing field for a particular odor, if the corresponding maximum mean firing rate was larger than the 95th percentile of the shuffled distribution. The time bin of that maximal rate was considered the cell’s field time bin. Cells with firing fields for both initiating odors were considered non-odor-specific. For these cells, if their field time bins for each odor were closer than 1 s, then their final field was considered the mean of the two time bins. If their field time bins were further apart than 1 s, then their field was the bin where the maximal average rate was higher. This only occurred in three PV cells (2.8% of all PV cells) and four SST cells (4.3%). For cells with no field, the time bin of their maximal average firing rate over all trials was computed.

The selectivity index (SI) of each cell was then computed as:

$${\rm{SI}}=\frac{{R}_{f}^{A}-{R}_{f}^{B}}{{R}_{f}^{A}+{R}_{f}^{B}}$$

where ${R}^{i}_{f}$ is the cell’s mean firing rate at its field (or at the time bin of maximal mean firing rate in the case of no-field cells) over all trials initiated by odor *i*. Cells that were initially detected as being odor-specific but had |SI| < 0.42 were considered as non-odor-specific. This criterion was implemented because many cells with similar firing rates in both odors were erroneously classified as odor-specific due to noisy spiking in other time bins as well. Chance odor selectivity for each cell was approximated by applying the above formula between two random sets of trials (of equal size on average), repeated 1,000 times. This distribution was concatenated across cells and averaged.

A cell’s firing rate change at different trial segments was computed as the trial-averaged relative change of its mean rate over the corresponding time bins, compared to its mean rate over the 0.8 s preceding the first odor (baseline).

#### Analysis across multiple days

The inflow of new odor fields at any session was computed as the percentage of cells of day *d* with an odor field that were also imaged in the previous session but did not exhibit a significant odor field then. Stable cells are defined as the percentage of cells of day *d* with an odor field that were also imaged in the previous session and did exhibit a significant odor field then too. The outflow of odor fields at a session was computed as the percentage of cells from the previous session with an odor field that were also imaged on the current one but did not retain an odor field.

For comparing between the firing rates or theta oscillations of tracked cells while the mouse was naive versus trained, we used the first trained session where the cell was imaged to compare against the one naive session where the cell was imaged (for one PV cell that was imaged on two naive sessions, we used the second session).

For cross-days correlations, firing rates over the odor-delay interval were *z*-scored for each trial and the Pearson correlation was computed for all pairs of trials between the two sessions. The mean correlation was computed.

The Spearman rank correlation was estimated for the relationship between mean pairwise correlations and distance between imaging sessions.

When estimating the evolution of collective activity from all cells across days, the one extra naive session in a single SST-Cre mouse was removed for analysis (so that all mice had two naive sessions). Performance was computed over the pooled trials from all trial blocks recorded with each cell. Rates, locomotion and theta amplitudes were averaged across the entire recording. For naive sessions, all performances were set to 50% corresponding to chance, as mice did not participate in the DNMS task.

Theta power refers to the mean theta amplitude over odor-delay interval and across trials.

#### Support vector machine and Bayesian decoding

For both decoding methods, trials from each cell were analyzed separately. The first two-thirds of all trials were used for training and the final one-third for decoding. Only recordings with at least ten trials total and at least two training trials of each odor were included. Rates over each trial were smoothed with a five-point moving-average window.

Binary SVM classifiers were created to decode the identity of the first odor in a trial. They were trained on the cell’s firing rates either across the first odor delivery or the entire odor-delay interval. Smoothed rates were *z*-scored over the corresponding bins in each trial. The classifier was trained using a radial basis function kernel with automatically selected scale factor and standardized predictors (function fitcsvm, MATLAB). Chance baseline for each cell was computed by shuffling the identity of both training and predicted odors with 500 repetitions and applying the SVM classifier on each repetition. Odor-decoding accuracy refers to the percentage of correctly decoded odors over all predicted trials.

Bayesian decoders were created to decode odor-specific timing in a trial, using a similar approach as reported in ref. ^5^. They were trained on the cell’s firing rates across the odor-delay interval. The rate of each trial was scaled to have a maximum value equal to the maximum over all trials. To decode the odor identity, we considered time space to be 2× odor-delay interval, by concatenating along the time axis, each cell’s mean rate over the two initiating odors (mean rate over odor A-initiated trials followed by mean rate over odor B-initiated trials). The decoder, trained by these concatenated firing rates, would thus predict a time point along a doubled time interval, 12-s long. If the time point was within the first half (0–6 s) it corresponded to that time point of an odor A trial, whereas if it was within the second half (6–12 s) it corresponded to the analogous time point of an odor B trial. Time bins with no activity in either group of trials were discarded.

The decoded time point ${\hat{T}}_{t}$ from the cell’s spiking at time bin *t* in a decoded trial is given by:

$${\hat{T}}_{t}=\begin{array}{c}\displaystyle{\mathop{\mathrm{argmax}}\limits_{t}}\end{array}\left(C\left(s\left(t\right),t\right)K(t)\frac{\tau{{R}_{m}\left(t\right)}^{s\left(t\right)}}{s\left(t\right)!}{e}^{-\tau{R}_{m}\left(t\right)}{e}^{\frac{{|t-{\hat{T}}_{t-1}|}^{2}}{2{\sigma}^{2}}}\right)$$

where *s(t)* is the number of spikes at time bin *t*, *R*_m_*(t)* is the cell’s mean firing rate across odor A trials concatenated with that across odor B trials, at time bin *t*, *τ* is the bin duration (100 ms) and *K* is the probability of being at time bin *t* of a trial initiated by a particular odor (proportional to the ratio of trials of that odor over all training trials) and *C(t)* is a probability normalization factor. The last term functions as a continuity constraint, limiting the decoded time bin to a relative proximity to the previous one, with *σ* = 3 s. Chance baselines were computed by randomly circularly shifting the time bins of each trial to be decoded, with 500 repetitions, and decoding the shifted trials for each repetition. Decoded time error refers to the mean absolute time distance between a given time point and the decoded one, irrespective of the initiating odor. It thus functions as a measure of the time-information carried by the cell. Odor-decoding accuracy refers to the percentage of correct odor decoding at any given time point (decoded time bin being in the correct half of extended time axis).

#### On, off trials

Locomotion and ΔF/Fs were smoothed with first-order Savitzky–Golay filter. Rates were smoothed with a five-point moving-average window. For each cell, all three measures were normalized by the maximum across trials of their mean-per-trial across the odor-delay interval.

### Processing and spike sorting of electrophysiological data

Spikes were sorted offline using Kilosort2 (ref. ^67^) with default parameters and criteria for single-unit clustering. Manual curation of labeled units included merging and splitting units based on quality metrics using Phy2.0 (https://github.com/kwikteam/phy). A putative single unit had to meet the following conditions: (1) the proportion of refractory violations must be <5%; (2) spike amplitudes throughout the recording distributed normally with 95% of spikes separated from the noise source; and (3) mean firing rate >0.5 Hz.

LFPs were downsampled to 2.5 kHz and 60 Hz noise was removed, together with 120 and 180 Hz harmonics, using second-order Butterworth IIR bandstop filters. The channel corresponding to CA1 pyramidal layer was manually estimated based on where theta phase reversal (assessed visually) occurred during locomotion as well as where ripple amplitudes were highest and were combined with sharp waves in deeper layers. Ripples were detected, as reported in ref. ^68^, when the amplitude (binned root mean square) of 120–200 Hz bandpassed LFPs exceeded 10 s.d. and ripple boundaries were set when the amplitude dropped below 2 s.d. Ripples lasting <40 ms were discarded and consecutive ones <50 ms apart were concatenated. The theta- and ripple-based criteria pointed to similar channels. Units were assigned to channels yielding highest action potential amplitude. Units assigned to channels within 100 µm from the CA1 channel were assumed to be within the CA1 pyramidal layer and stratum oriens and were kept for further analysis. For bilateral recordings, this was conducted independently for each probe.

Units were clustered into putative pyramidal cells (broad) and interneurons (narrow spiking)^69^. The amplitudes and time points of each unit’s mean waveform peak and trough were computed, together with the unit’s mean firing rate. Principal-component analysis was performed on (1) the peak–trough time distance; (2) the peak–trough amplitude ratio; and (3) the mean firing rate across no-stimulation trials, of pooled units of all animals after *z*-scoring them. Scores from the three principal components were split into two clusters using *k*-means with squared Euclidean distance measure and 100 clustering repeats, yielding two well-separated clusters with one containing ~16% of all units with shorter peak–trough distances and ratios and higher firing rates. Narrow spiking units in that cluster are considered as putative interneurons, whereas those in the opposite cluster are considered as putative pyramidal cells. Sparse spiking cells with mean rates across no-stimulation trials <0.5 Hz were discarded.

### Analysis of electrophysiological data

Firing rates were computed over 10-ms nonoverlapping bins. Odor responses were quantified as smoothed firing rates (with a 50-ms window Gaussian-weighted average), normalized by baseline (mean rate across the 1 s before the first odor in a trial) and averaged across the first odor cue. Example raster plots depict pooled trials with no stimulation.

Detection of significant fields followed the algorithm reported in ref. ^5^ but was applied to firing rates, smoothed with a Gaussian-weighted average (300-ms window) and *z*-scored per trial. For plotting, smoothed sequence-cell rates were averaged over corresponding trials and were normalized by their maximum value over the odor-delay interval. Odor selectivity indexes were computed across pooled trials without stimulation. They were quantified as before with positive value for the preferred odor, irrespective of odor identity. Changes in firing rates by optogenetic stimulation, were assessed only across trials of the trial block(s) with the corresponding stimulation protocol, comparing trials with and without stimulation. This method avoided comparisons being affected by unit drift, slow changes in unit activity or by any spiking influences by preceding stimulation protocols.

### Two-photon calcium imaging and data processing and analysis

The recording protocol and calcium data processing was described in ref. ^5^. In brief, two-photon imaging sessions started ~2–3 weeks post-surgery while mice were either in DNMS training or were well trained. A single fixed FOV was imaged every day for each mouse. A total of 58 recording sessions from 11 mice were included. Calcium data were processed in MATLAB using a custom-built pipeline based on the CaImAn package^62^. GABAergic neurons were identified based on their static tdTomato fluorescence recorded for 500 frames at the beginning of each session on the red-channel photomultiplier tube, together with the functional (green) channel. The ROIs from each channel were then registered and ROIs from the green channel that matched those from the red were discarded, so that only pyramidal cell activity from the green channel was analyzed further.

Pooled cells were sorted based on the mean value of the trial-averaged deconvolved signal, over the first odor in each trial. The trial-averaged deconvolved signal was then *z*-scored. The mean value of the *z*-scored signal over the first odor was used as the cell’s odor response. Detection of significant fields followed the algorithm reported in ref. ^5^ but was applied to the raw deconvolved signal here, instead of its binned version.

When plotting pooled field-cell spiking (Fig. 6f), average deconvolved signals over preferred or nonpreferred trials were normalized by the cell’s mean signal at its field. Example deconvolved signals (Fig. 6b) as well as trial-average ones were smoothed with a five-point moving-average window for plotting.

### Statistics and reproducibility

No statistical methods were used to predetermine sample sizes. Sample sizes of mice and recorded neurons are similar to those reported in in vivo voltage imaging studies (for example see refs. ^58,59^), calcium imaging (for example see refs. ^5,56^) and time-cell studies (for example see refs. ^7–9^). They were based on reliably measuring experimental parameters through large numbers of neurons, while minimizing the number of experimental animals. Mice with poor viral expression of ASAP3 or GCaMP6f or mice with problematic craniotomies were excluded before experiments. ASAP3-expressing cells with poor signal-to-noise ratio that did not generate detectable spikes were not recorded. One mouse with problematic Neuropixel probe placement was excluded from the analysis. Mice were not split into groups. Experiments were not randomized and the Investigators were not blinded to allocation during experiments and outcome assessment.

### Reporting summary

Further information on research design is available in the Nature Portfolio Reporting Summary linked to this article.

## Online content

Any methods, additional references, Nature Portfolio reporting summaries, source data, extended data, supplementary information, acknowledgements, peer review information; details of author contributions and competing interests; and statements of data and code availability are available at 10.1038/s41593-025-02016-y.

## Supplementary information

Reporting Summary

## Data Availability

Pooled and processed voltage imaging datasets are available at Zenodo at 10.5281/zenodo.15299606 (ref. ^70^). Unprocessed voltage imaging data from each individual session, as well as electrophysiology and calcium imaging datasets, are available from the corresponding authors upon reasonable request.

## Code availability

All custom-written code (MATLAB 2016b) is available at https://github.com/jtaxidis-sickkids/Taxidis-et-al.-2025.
