# Supplementary material for: Voltage imaging reveals hippocampal inhibitory dynamics shaping pyramidal memory-encoding sequences
Source: Nat Neurosci. 2025 Jul 22;28(9):1946–58. doi: 10.1038/s41593-025-02016-y (PMC12411278; doi:10.1038/s41593-025-02016-y)
Supplement: Supplementary file 1 — Reporting Summary [file 41593_2025_2016_MOESM1_ESM.pdf]

Corresponding author(s): Jiannis Taxidis, Peyman Golshani

Last updated by author(s): Apr 23, 2025

Reporting Summary

Nature Portfolio wishes to improve the reproducibility of the work that we publish. This form provides structure for consistency and transparency in reporting. For further information on Nature Portfolio policies, see our Editorial Policies and the Editorial Policy Checklist.

Statistics

For all statistical analyses, confirm that the following items are present in the figure legend, table legend, main text, or Methods section.

n/a	Confirmed	
☐	☒	The exact sample size (*n*) for each experimental group/condition, given as a discrete number and unit of measurement
☐	☒	A statement on whether measurements were taken from distinct samples or whether the same sample was measured repeatedly
☐	☒	The statistical test(s) used AND whether they are one- or two-sided<br>*Only common tests should be described solely by name; describe more complex techniques in the Methods section.*
☐	☒	A description of all covariates tested
☐	☒	A description of any assumptions or corrections, such as tests of normality and adjustment for multiple comparisons
☐	☒	A full description of the statistical parameters including central tendency (e.g. means) or other basic estimates (e.g. regression coefficient) AND variation (e.g. standard deviation) or associated estimates of uncertainty (e.g. confidence intervals)
☐	☒	For null hypothesis testing, the test statistic (e.g. *F*, *t*, *r*) with confidence intervals, effect sizes, degrees of freedom and *P* value noted<br>*Give P values as exact values whenever suitable.*
☐	☒	For Bayesian analysis, information on the choice of priors and Markov chain Monte Carlo settings
☒	☐	For hierarchical and complex designs, identification of the appropriate level for tests and full reporting of outcomes
☐	☒	Estimates of effect sizes (e.g. Cohen's *d*, Pearson's *r*), indicating how they were calculated

Our web collection on statistics for biologists contains articles on many of the points above.

Software and code

Policy information about availability of computer code

Data collection	Voltage imaging data were collected using the HCImage Live v4.5.1 software (Hamamatsu). Calcium data were recorded using the SciScan software (Scientifica). Neuropixel data were collected with Spike GLX. All other data collection was performed using custom written code in Matlab (2016b). Time stamps for recording data points and for behavioral data were generated with WinEDR (Strathclyde Electrophysiology)
Data analysis	Initial processing of voltage imaging data was performed with a modified version of the Volpy numerical pipeline (Cai et al. PLOS Comp Biol 2021) in Python. Initial processing of Neuropixel data was performed with Kilosort2 and Phy2.0. Initial processing of calcium data was performed with a custom-built pipeline based on CaImAn (Giovannucci et al. Elife 2019) in Matlab (as in Taxidis et al. Neuron 2020). All other data analyses were performed using custom written code in Matlab (2016b)<br><br>All custom-written Matlab code is available at https://github.com/jtaxidis-sickkids/Taxidis-et-al.-2025

For manuscripts utilizing custom algorithms or software that are central to the research but not yet described in published literature, software must be made available to editors and reviewers. We strongly encourage code deposition in a community repository (e.g. GitHub). See the Nature Portfolio guidelines for submitting code & software for further information.

Data

Policy information about availability of data

All manuscripts must include a data availability statement. This statement should provide the following information, where applicable:

- Accession codes, unique identifiers, or web links for publicly available datasets
- A description of any restrictions on data availability
- For clinical datasets or third party data, please ensure that the statement adheres to our policy

Pooled, processed, voltage imaging datasets are available at 10.5281/zenodo.15299606. Unprocessed voltage imaging data from each individual session, as well as electrophysiology and calcium imaging datasets, are available by the corresponding authors upon reasonable request. This is due to the large file sizes for most generated datasets and the complexity and diversity of experimental conditions and file-types (e.g. pairing voltage imaging files with behavior files and timestamp files for each recording session). Our lab can best help with accessing and organizing data according to specific requests.

Research involving human participants, their data, or biological material

Policy information about studies with human participants or human data. See also policy information about sex, gender (identity/presentation), and sexual orientation and race, ethnicity and racism.

Reporting on sex and gender	N/A
Reporting on race, ethnicity, or other socially relevant groupings	N/A
Population characteristics	N/A
Recruitment	N/A
Ethics oversight	N/A

Note that full information on the approval of the study protocol must also be provided in the manuscript.

Field-specific reporting

Please select the one below that is the best fit for your research. If you are not sure, read the appropriate sections before making your selection.

☒ Life sciences ☐ Behavioural & social sciences ☐ Ecological, evolutionary & environmental sciences

For a reference copy of the document with all sections, see nature.com/documents/nr-reporting-summary-flat.pdf

Life sciences study design

All studies must disclose on these points even when the disclosure is negative.

Sample size	No statistical methods were used to predetermine sample sizes. Sample sizes of mice and recorded neurons are described in detail in Methods and are similar to those typically reported by us and other labs, in the fields of in vivo voltage imaging (e.g. Adam et al. 2019, Fan et al. 2023), calcium imaging (e.g. Taxidis et al. 2020, Dorian et al. 2024) and time cell analysis (e.g. Pastalkova et al. 2008, MacDonald et al. 2011,2013). They were based on reliably measuring experimental parameters through a large number of neurons, while minimizing the number of experimental animals to remain in compliance with ethical guidelines.
Data exclusions	Mice with poor viral expression of ASAP3 or GCaMP6f accordingly or mice with problematic craniotomies were excluded prior to experiments. ASAP3-expressing cells with poor signal-to-noise ratio that did not generate detectable spikes were not recorded. Recorded trials where the cell had become photo-bleached (no spikes or very poor spikes detected for all ensuing trials) were excluded from analysis as described in the Methods section of the manuscript. One mouse with problematic Neuropixel probe placement was excluded from the analysis.
Replication	Each mouse was recorded separately during each imaging or electrophysiology session. For voltage imaging, most cells were also recorded separately as described in Methods. Each recording session was processed separately. Successful replication is inferred by the fact that similar results were observed when comparing across neurons recorded in a mouse, as well as when comparing across separate mice and when pooling data from all corresponding mice across each experiment.
Randomization	Mice were not split into experimental groups. All DNMS trials were randomized so that each mouse received a random combination of odors in each trial, during both training and recording sessions. For data analyses, randomized shuffling of data or chance baselines were created as described in Methods, using standard algotirhtms in Matlab.
Blinding	Experiments and analyses were not performed blind since mice were not split to experimental groups.

Reporting for specific materials, systems and methods

We require information from authors about some types of materials, experimental systems and methods used in many studies. Here, indicate whether each material, system or method listed is relevant to your study. If you are not sure if a list item applies to your research, read the appropriate section before selecting a response.

Materials & experimental systems

n/a	Involved in the study
☒	☐ Antibodies
☒	☐ Eukaryotic cell lines
☒	☐ Palaeontology and archaeology
☐	☒ Animals and other organisms
☒	☐ Clinical data
☒	☐ Dual use research of concern
☒	☐ Plants

Methods

n/a	Involved in the study
☒	☐ ChIP-seq
☒	☐ Flow cytometry
☒	☐ MRI-based neuroimaging

Animals and other research organisms

Policy information about studies involving animals; ARRIVE guidelines recommended for reporting animal research, and Sex and Gender in Research

Laboratory animals	All animals used were adult mice (8-31 weeks old) of the following strains: PV-Cre, SST-IRES-Cre, Gad2-Cre:Ai9, Gad2-Cre:Ai14 and WT as described in detail in the Methods section. All animals were group housed (2-5 per cage) in a vivarium with a 12 h light/dark cycle, temperature of 20–26°C (68–79°F), relative humidity 30%–70% and with food and water available ad libitum (except when water restricted). All experimental protocols were approved by the Chancellor's Animal Research Committee of the University of California, Los Angeles, in accordance with the National Institute of Health (NIH) guidelines.
Wild animals	No wild animals were used in the study.
Reporting on sex	29 male and 2 female mice were used in total.
Field-collected samples	No field collected samples were used in the study.
Ethics oversight	All experimental protocols were approved by the Chancellor's Animal Research Committee of the University of California, Los Angeles, in accordance with NIH guidelines.

Note that full information on the approval of the study protocol must also be provided in the manuscript.
